# Supplementary material for: Enhancing nonlinear transcriptome- and proteome-wide association studies via trait imputation with applications to Alzheimer’s disease
Source: PLoS Genet. 2025 Apr 10;21(4):e1011659. doi: 10.1371/journal.pgen.1011659 (PMC12040266; doi:10.1371/journal.pgen.1011659)
Supplement: S1 Text — (PDF) [file pgen.1011659.s001.pdf]

Supplementary materials: Enhancing nonlinear TWAS/PWAS performance via trait imputation with applications to Alzheimer's disease

Ruoyu He[1,2], Jingchen Ren[1,2], Mykhaylo M. Malakhov[2], and Wei Pan[2,*]

[1]School of Statistics, University of Minnesota, Minneapolis, MN, 55455

[2]Division of Biostatistics and Health Data Science, School of Public Health, University of Minnesota, Minneapolis, MN, 55455

[*]Corresponding author. Email: panxx014@umn.edu

Contents

1 GWAS marginal effects calculated from LS-imputed AD on UKB were similar to publicly available GWAS 2

2 GWAS marginal effects based on AD Proxy were biased 8

2.0.1 LS-imputation faithfully recovered the genetic landscape captured by the training GWAS data 11

3 Differently imputed AD traits were very weakly correlated 16

4 Simulation 16

4.1 Setup 16

4.2 DeLIVR trained with the LS-imputed data controlled the Type I error rate at the nominal level and improved power 19

5 TWAS/PWAS analysis with different data QC processes and stage 1 models 20

5.0.1 Stage 2 datasets 20

5.1 DeLIVR uniquely identified genes/proteins related to AD using imputed AD status 21

6 TWAS/PWAS results with significance cutoff 1×10^{-3} 21

1 GWAS marginal effects calculated from LS-imputed AD on UKB were similar to publicly available GWAS

In this section, we compare the estimated marginal effect sizes, standard errors (SEs), and $-\log_{10}(p)$-values obtained from the training GWAS data (EADB or IGAP) with those derived from the LS-imputed traits calculated on the UKB (test) data. This analysis mirrors the comparison detailed in Section 3.1.1 of the main text but includes additional results not presented there. Specifically, we provide comparisons for all batch sizes considered and include plots that incorporate the "outlier" variants excluded from the figures in the main text.

Fig A compares the estimated marginal effect sizes from the EADB GWAS training data with those calculated using LS-imputed AD status on the UKB test data. The left-hand plots—(a), (c), and (e)—display all $p = 70,000$ SNPs used for imputation. Notably, three

SNPs exhibit significantly larger effect sizes (in absolute value) compared to the other SNPs. To enhance visualization, these three "outliers" were excluded, and the plots were redrawn on the right-hand side—(b), (d), and (f). These revised plots depict the same results as (a), (c), and (e) but with the outliers removed. The Pearson correlation coefficients for the original plots—(a), (c), and (e)—are 0.995, 0.997, and 0.998, respectively.

Fig B compares the SEs obtained from the EADB GWAS training data with those calculated using LS-imputed AD status on the UKB test data. These SEs correspond to the effect size estimates shown in the previous figure for the same set of $p = 70,000$ SNPs. As before, the plots in the left-hand column display all variants. Notably, four variants exhibit substantially larger SEs in EADB compared to the other variants. To improve visualization, these four "outliers" were excluded, and the plots were redrawn in the right-hand column. Specifically, plots (b), (d), and (f) present the same results as (a), (c), and (e) but with the four outlying variants removed. The Pearson correlation coefficients for the original plots—(a), (c), and (e)—are all 0.749.

Fig C compares the $-\log_{10}(p)$-values obtained from the EADB GWAS training data with those calculated using LS-imputed AD status on the UKB test data. These $-\log_{10}(p)$-values correspond to the effect sizes and SEs presented in the previous two figures. The Pearson correlation coefficients for plots (a), (b), and (c) are 0.941, 0.963, and 0.972, respectively.

The next three figures present analogous results, using summary statistics from the IGAP GWAS. Fig D compares the estimated marginal effect sizes obtained from the IGAP GWAS training data with those calculated using LS-imputed AD status on the UKB test data. The figures display effect size estimates for all $p = 60,000$ variants used for imputation. Unlike the EADB GWAS, the IGAP GWAS does not contain any outliers, so only one set of three plots is shown. The Pearson correlation coefficients for plots (a), (b), and (c) are 0.992, 0.996, and 0.998, respectively.

Fig E compares the SEs obtained from the IGAP GWAS training data with those calculated using LS-imputed AD status on the UKB test data for the $p = 60,000$ variants used in imputation. Specifically, this figure presents the SEs corresponding to the effect sizes shown in the previous figure. The Pearson correlation coefficients for plots (a), (b), and (c) are all 0.922.

Fig F compares the $-\log_{10}(p)$-values obtained from the IGAP GWAS training data with those calculated using LS-imputed AD status on the UKB test data for the $p = 60,000$ variants used in imputation. Specifically, these plots display the $-\log_{10}(p)$-values for the same variants whose effect sizes and SEs were presented in the previous two figures. The Pearson correlation coefficients for plots (a), (b), and (c) are 0.831, 0.880, and 0.891, respectively.

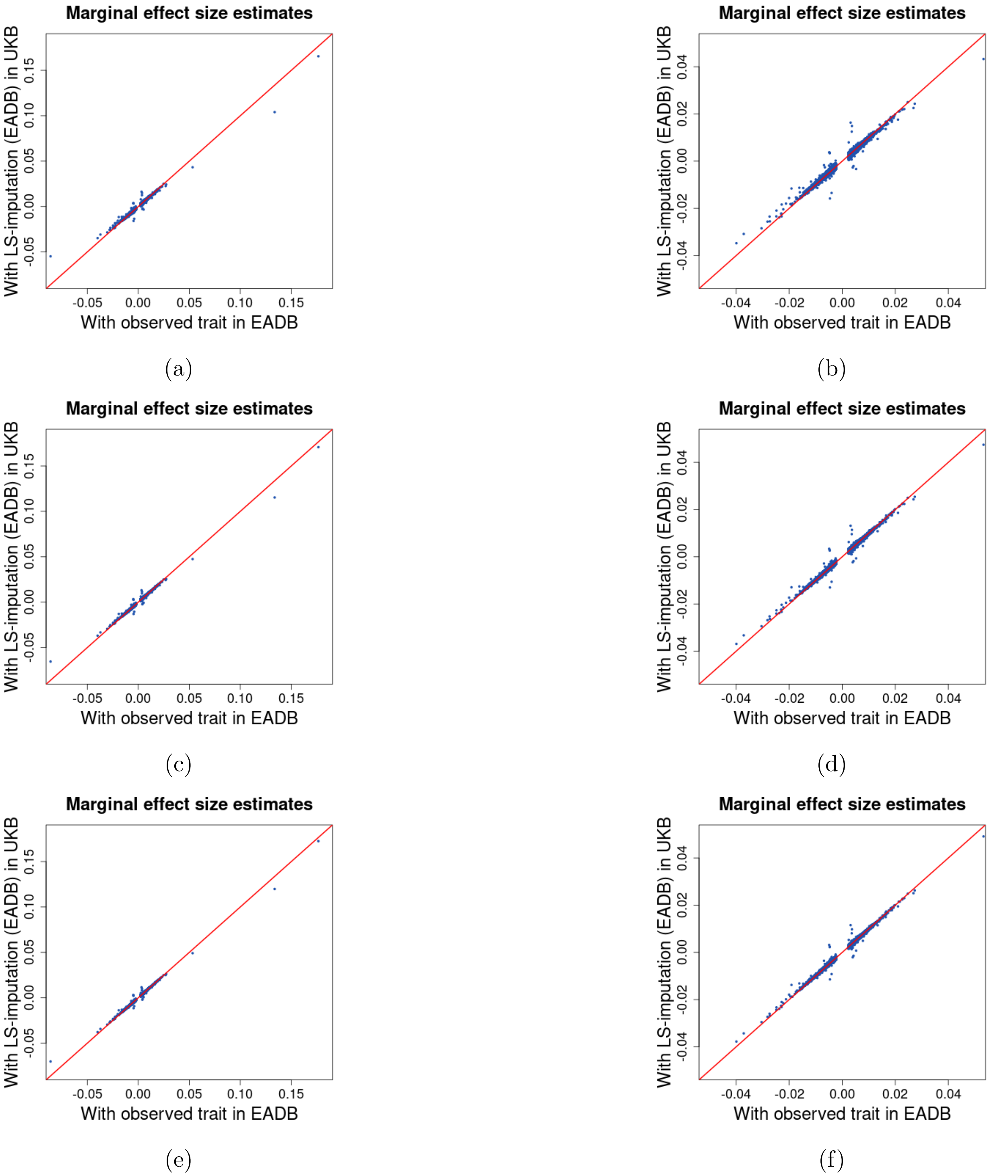

Figure A: Comparison of estimated marginal effect sizes obtained from the training data (EADB) with those calculated using LS-imputed AD status on the test data (UKB) for $p = 70,000$ SNPs. The number of samples per batch was (a)-(b): $m = 40,000$, (c)-(d): $m = 50,000$, (e)-(f): $m = 60,000$. (a),(c),(e) display the full results while (b),(d),(f) display the results with "outliers" removed.

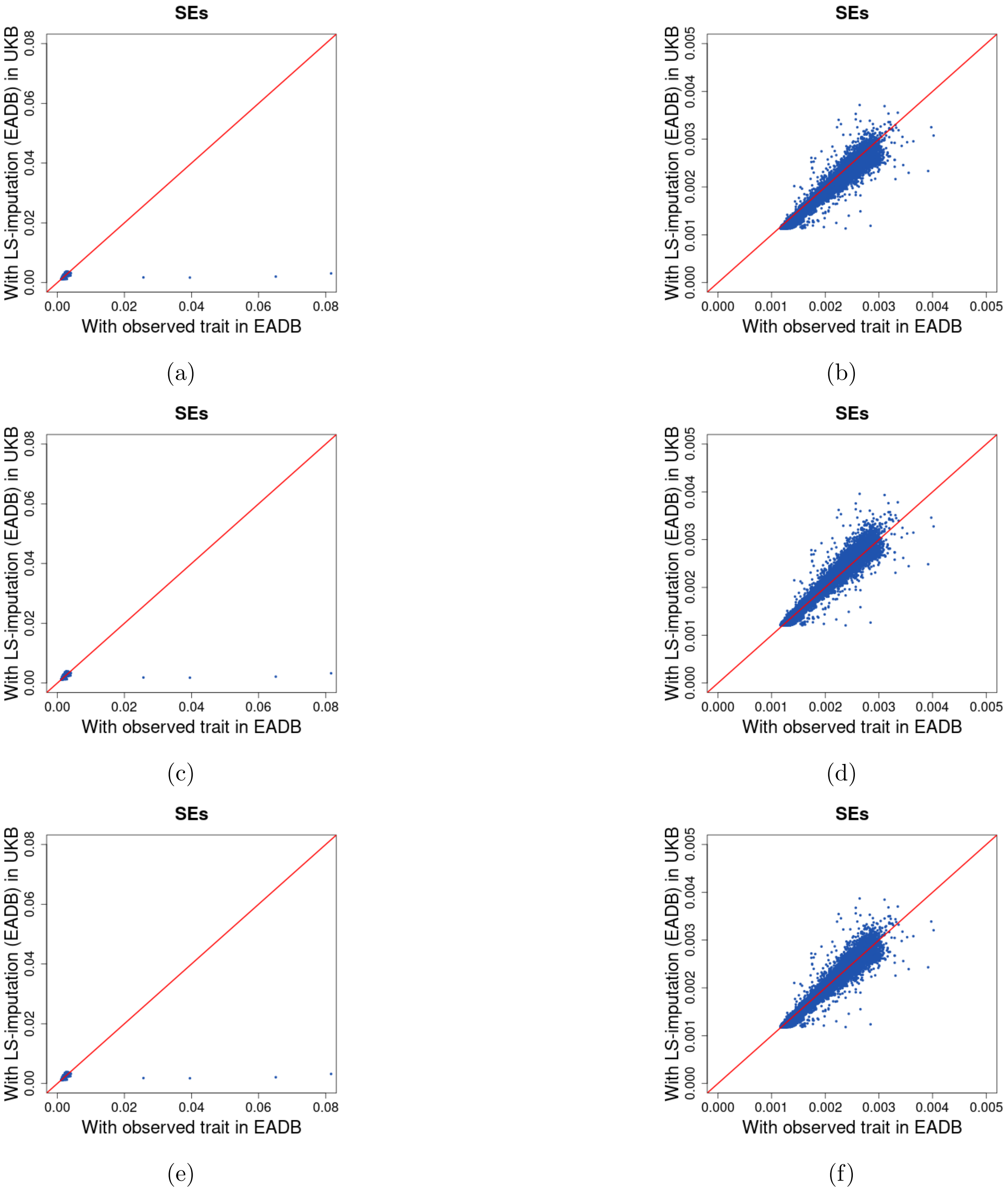

Figure B: Comparison of SEs obtained from the training data (EADB) with those calculated using LS-imputed AD status on the test data (UKB) for $p = 70,000$ SNPs. The number of samples per batch was (a)-(b): $m = 40,000$, (c)-(d): $m = 50,000$, (e)-(f): $m = 60,000$. (a),(c),(e) display the full results while (b),(d),(f) display the results with "outliers" removed.

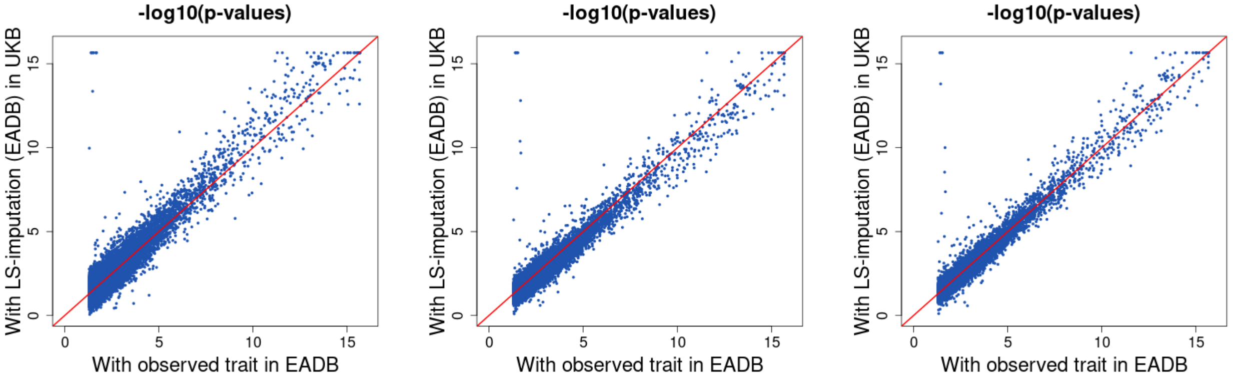

(a) (b) (c)

Figure C: Comparison of -log10(p-values) obtained from the training data (EADB) with those calculated using LS-imputed AD status on the test data (UKB) for $p = 70,000$ variants. The number of samples per batch was (a): $m = 40,000$, (b): $m = 50,000$, (c): $m = 60,000$.

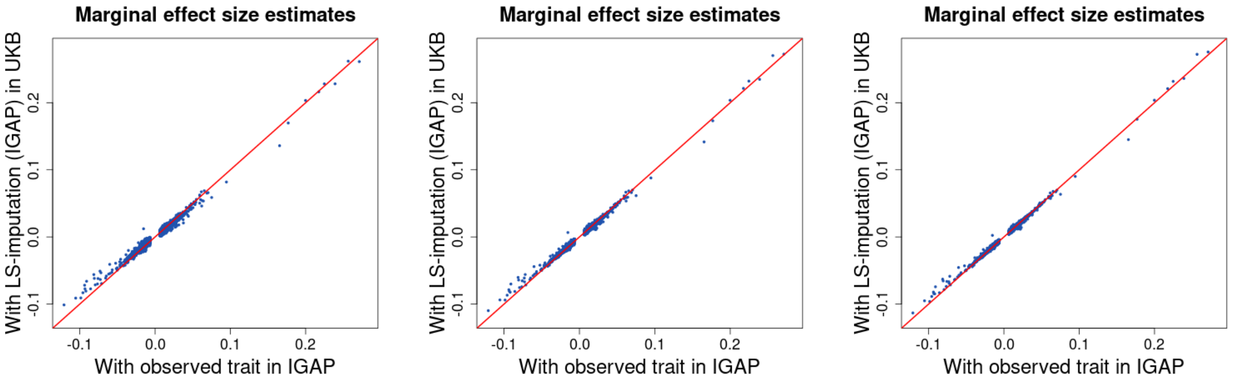

(a) (b) (c)

Figure D: Comparison of estimated marginal effect sizes obtained from the training data (IGAP) with those calculated using LS-imputed AD status on the test data (UKB) for $p = 60,000$ variants. The number of samples per batch was (a): $m = 30,000$, (b): $m = 40,000$, (c): $m = 50,000$.

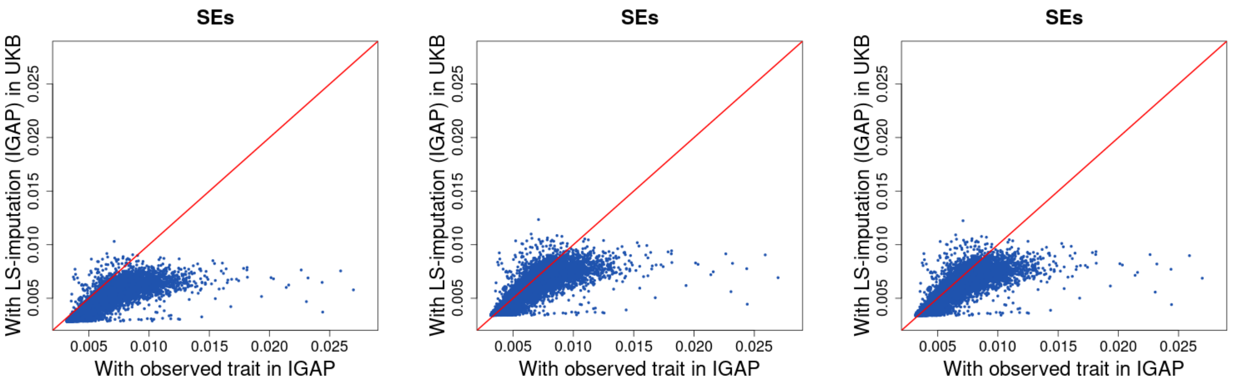

(a) (b) (c)

Figure E: Comparison of SEs obtained from the training data (IGAP) with those calculated using LS-imputed AD status on the test data (UKB) for $p = 60,000$ variants. The number of samples per batch was (a): $m = 30,000$, (b): $m = 40,000$, (c): $m = 50,000$.

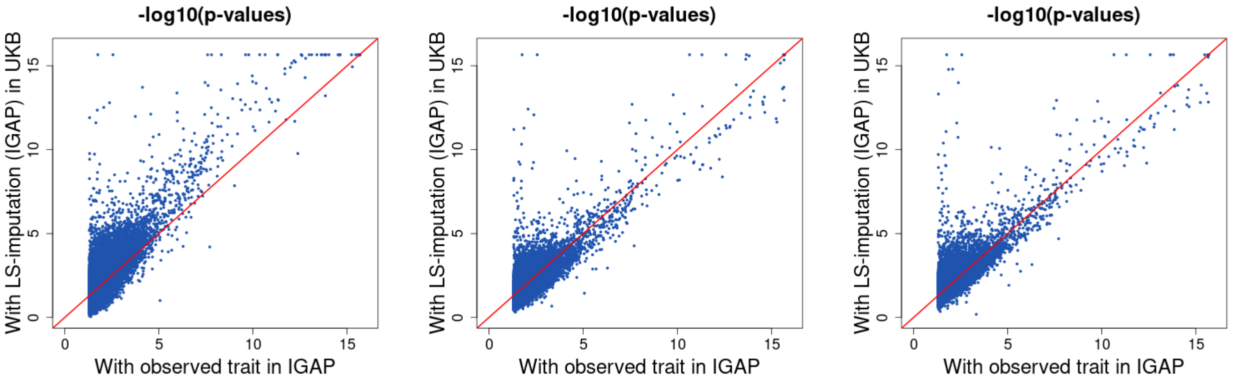

(a) (b) (c)

Figure F: Comparison of -log10(p-values) obtained from the training data (IGAP) with those calculated using LS-imputed AD status on the test data (UKB) for $p = 60,000$ variants. The number of samples per batch was (a): $m = 30,000$, (b): $m = 40,000$, (c): $m = 50,000$.

2 GWAS marginal effects based on AD Proxy were biased

In this section, we compare the estimated marginal effect sizes, standard errors (SEs), and $-\log_{10}(p)$-values calculated from AD status imputed using the AD Proxy method with those calculated from AD status imputed using the LS-imputation method. These comparisons are mentioned in Section 3.1.1 of the main text, but here we present results for all UKB batch sizes considered. Additionally, we include the full data in cases where outliers were previously removed.

Fig G compares the estimated marginal effect sizes calculated from AD Proxy with those calculated from LS-imputed AD status using EADB as the training data. Both methods were used to impute AD status for UKB individuals, with $p = 70,000$ variants included in LS-imputation. Notably, three SNPs exhibit much larger effect sizes compared to the rest. The left-hand plots—(a), (c), and (e)—display all 70,000 SNPs, while the right-hand plots—(b), (d), and (f)—show the same results with the three "outliers" removed. The Pearson correlation coefficients for plots (a), (c), and (e) are 0.805, 0.804, and 0.804, respectively.

Fig H compares the SEs calculated from AD Proxy with those calculated from LS-imputed AD status using EADB as the training data. Both methods were used to impute AD status for UKB individuals, with $p = 70,000$ variants included in LS-imputation. Specifically, this figure presents the SEs corresponding to the effect sizes shown in the previous figure. The Pearson correlation coefficients for plots (a), (b), and (c) are all 0.999.

Fig I compares the $-\log_{10}(p)$-values calculated from AD Proxy with those calculated from LS-imputed AD status using EADB as the training data. Both methods were used to impute AD status for UKB individuals, with $p = 70,000$ variants included in LS-imputation. Specifically, this figure presents the $-\log_{10}(p)$-values for the same variants whose effect sizes and standard errors were shown in the previous two figures. The Pearson correlation coefficients for plots (a), (b), and (c) are 0.505, 0.514, and 0.513, respectively.

The next three figures present analogous results, using IGAP GWAS data. Fig J compares the estimated marginal effect sizes calculated from AD Proxy with those calculated from LS-imputed AD status using IGAP as the training data. Both methods were used to impute AD status for UKB individuals, with $p = 60,000$ variants included in LS-imputation. Notably, these results do not contain any outliers, so only one plot is shown for each batch size. The Pearson correlation coefficients for plots (a), (b), and (c) are 0.155, 0.150, and 0.149, respectively.

Fig K compares the SEs calculated from AD Proxy with those calculated from LS-imputed AD status using IGAP as the training data. Both methods were used to impute AD status

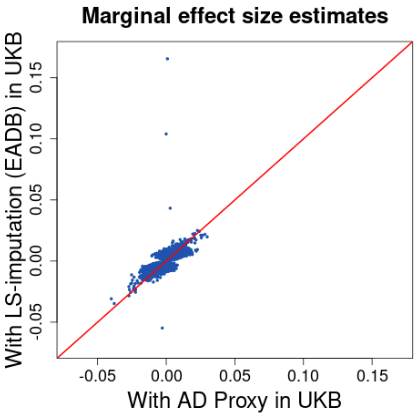

(a)

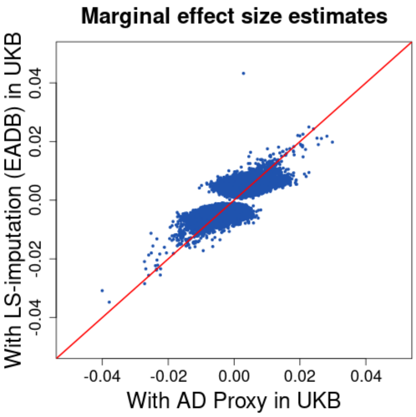

(b)

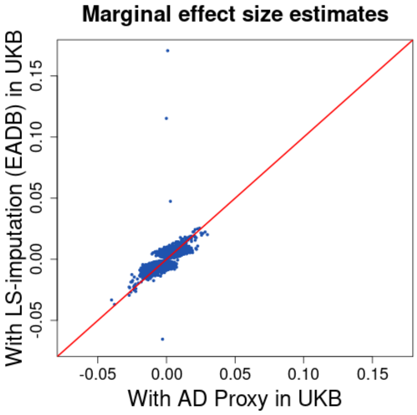

(c)

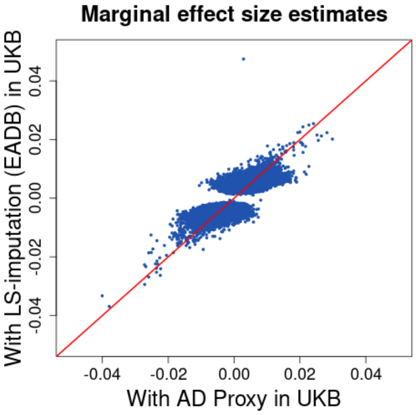

(d)

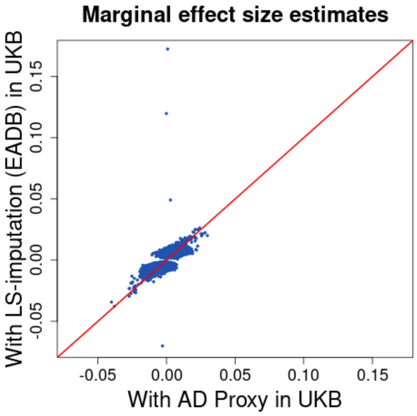

(e)

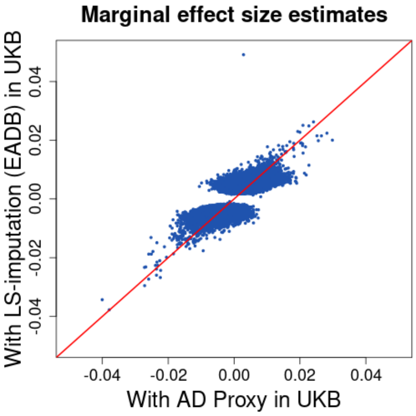

(f)

Figure G: Comparison of estimated marginal effect sizes calculated from AD Proxy with those calculated from LS-imputed AD status for $p = 70,000$ variants. EADB summary statistics were used as training data for LS-imputation. The number of samples per batch was (a)-(b): $m = 40,000$, (c)-(d): $m = 50,000$, (e)-(f): $m = 60,000$. (a),(c),(e) display all variants while (b),(d),(f) have "outliers" removed.

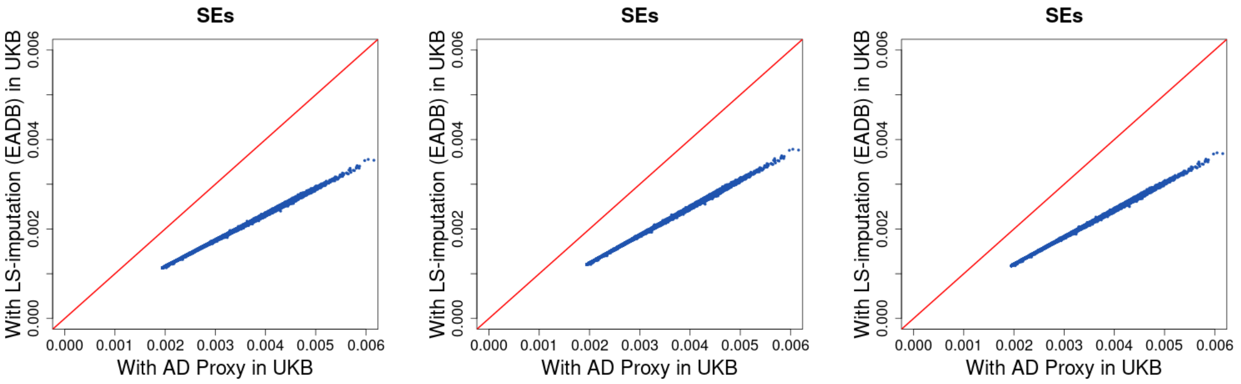

(a) (b) (c)

Figure H: Comparison of SEs calculated from AD Proxy with those calculated from LS-imputed AD status for $p = 70,000$ variants. EADB summary statistics were used as training data for LS-imputation. The number of samples per batch was (a): $m = 40,000$, (b): $m = 50,000$, (c): $m = 60,000$.

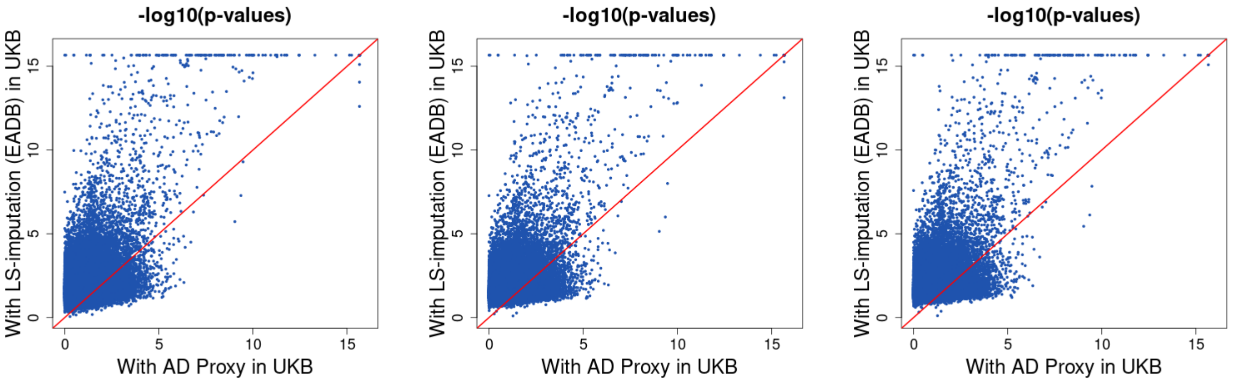

(a) (b) (c)

Figure I: Comparison of -log10(p-values) calculated from AD Proxy with those calculated from LS-imputed AD status for $p = 70,000$ variants. EADB summary statistics were used as training data for LS-imputation. The number of samples per batch was (a): $m = 40,000$, (b): $m = 50,000$, (c): $m = 60,000$.

for UKB individuals, with $p = 60,000$ variants included in LS-imputation. Specifically, this figure presents the SEs corresponding to the effect sizes shown in the previous figure. The Pearson correlation coefficients for plots (a), (b), and (c) are all 0.999.

Fig L compares the $-\log_{10}(p)$-values calculated from AD Proxy with those calculated from LS-imputed AD status using IGAP as the training data. Both methods were used to impute AD status for UKB individuals, with $p = 60,000$ variants included in LS-imputation. Specifically, this figure presents the $-\log_{10}(p)$-values for the same variants whose effect sizes and standard errors were shown in the previous two figures. The Pearson correlation coefficients for plots (a), (b), and (c) are 0.387, 0.409, and 0.407, respectively.

2.0.1 LS-imputation faithfully recovered the genetic landscape captured by the training GWAS data

In addition to LS-imputation, we conducted GWAS analyses using AD status obtained through the other methods described in Section 2 of the main text. Specifically, we considered clinically diagnosed AD, AD Proxy, AD Proxy2, AD status imputed with PRS-CS, and AD status imputed with LDpred2. All imputations were performed for individuals in the UKB dataset.

Fig M compares the GWAS results obtained from several imputation methods trained on IGAP summary statistics. Notably, the distribution of significant variants identified using LS-imputed AD and AD Proxy was more similar to the distribution of significant variants identified in the original IGAP GWAS data compared to other imputation methods. AD Proxy was more informative on chromosome 6, whereas LS-imputation was more informative on other chromosomes. Clinically diagnosed AD was overly conservative, while an excessive number of significant variants were identified when using AD status imputed with PRS-CS or LDpred2.

Fig N similarly presents GWAS results from each imputation method, trained on EADB summary statistics. Due to the larger sample size of EADB, more significant variants were identified in the EADB GWAS compared to IGAP, and this was reflected in the imputed AD GWAS results. All GWAS-based imputation methods identified more significant loci when trained on EADB summary data compared to IGAP summary data, with LS-imputation showing the greatest similarity to the original EADB GWAS results. It is important to note that AD Proxy and AD Proxy2 did not use any external GWAS information (neither IGAP nor EADB). Compared to LS-imputation with EADB as the training data, both AD Proxy and AD Proxy2 identified fewer significant loci, suggesting that LS-imputation may be a better alternative when trained on more informative GWAS summary statistics.

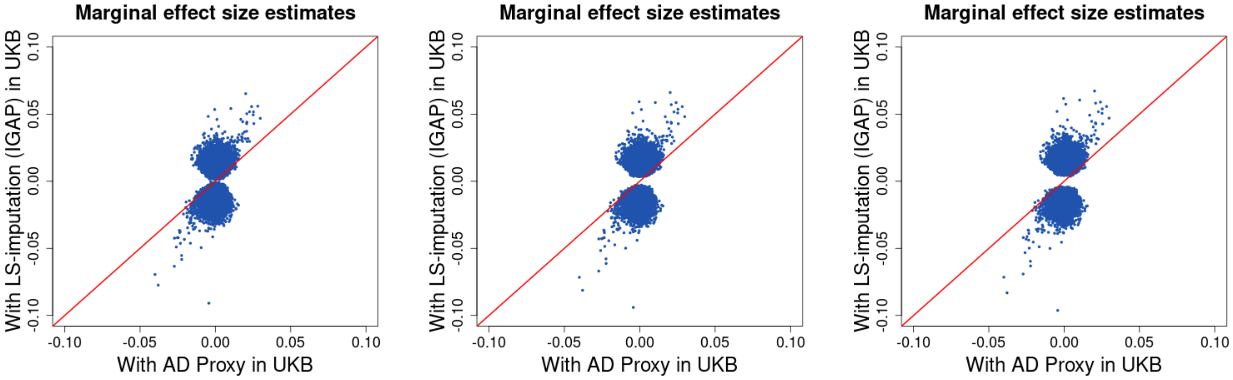

(a) (b) (c)

Figure J: Comparison of estimated marginal effect sizes calculated from AD Proxy with those calculated from LS-imputed AD status for $p = 60,000$ variants. IGAP summary statistics were used as training data for LS-imputation. The number of samples per batch was (a): $m = 30,000$, (b): $m = 40,000$, (c): $m = 50,000$.

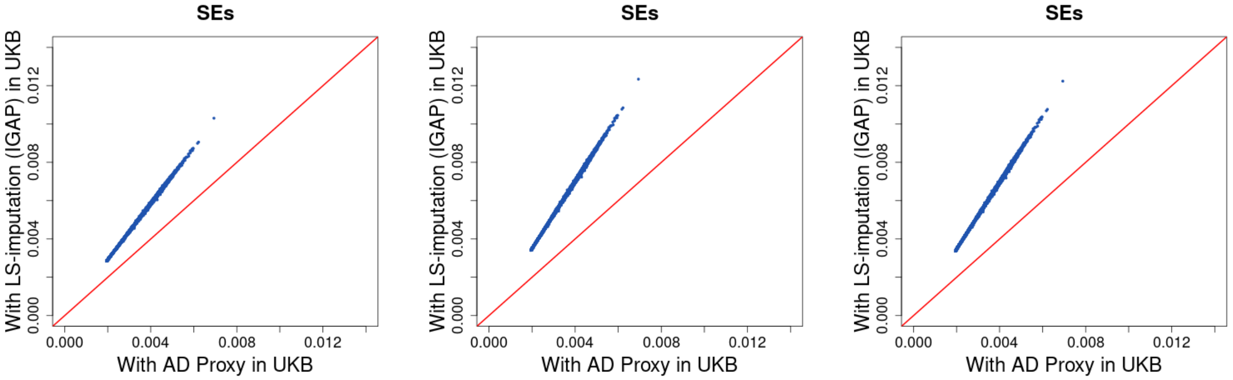

(a) (b) (c)

Figure K: Comparison of SEs calculated from AD Proxy with those calculated from LS-imputed AD status for $p = 60,000$ variants. IGAP summary statistics were used as training data for LS-imputation. The number of samples per batch was (a): $m = 30,000$, (b): $m = 40,000$, (c): $m = 50,000$.

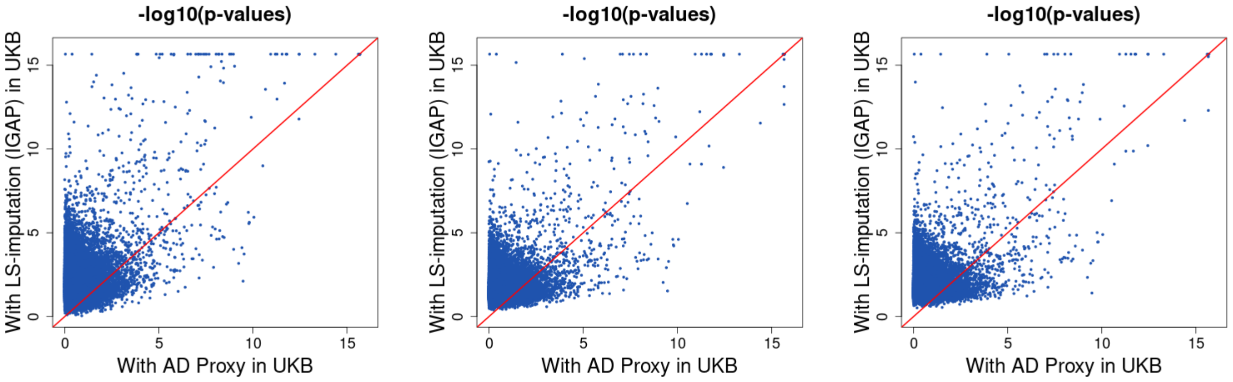

(a) (b) (c)

Figure L: Comparison of -log10(p-values) calculated from AD Proxy with those calculated from LS-imputed AD status for $p = 60,000$ variants. IGAP summary statistics were used as training data for LS-imputation. The number of samples per batch was (a): $m = 30,000$, (b): $m = 40,000$, (c): $m = 50,000$.

Since the comparisons of the Manhattan plots were based purely on visual cues, we present Venn diagrams of the significant SNPs identified by different GWAS analyses in Fig O. Five different models were considered, excluding those that used PRS-imputed traits. Notably, LS-imputation has the highest number of overlaps with the GWAS study used for imputation (IGAP or EADB). All analyses show the same number of overlaps when using Diagnosed AD status. These results suggest that LS-imputed traits may be more informative for capturing the genetic landscape of AD compared to AD Proxy and AD Proxy2, as LS-imputation leverages information from external GWAS studies.

In summary, the distribution of significant SNPs identified with LS-imputed AD status (using either IGAP or EADB summary statistics as the training data) is similar to that obtained from the corresponding GWAS results (IGAP or EADB). LS-imputation appears to be more informative than AD Proxy and AD Proxy2, depending on the training GWAS data. However, GWAS analyses performed using AD status imputed by PRS-CS and LDpred2 revealed an inflated number of significant SNPs. This observation aligns with the findings of Ren et al. (2023), who noted that any variants included in a PRS-CS (or any other PRS) model, along with those in linkage disequilibrium (LD) with them, are deemed significant given a sufficiently large sample size.

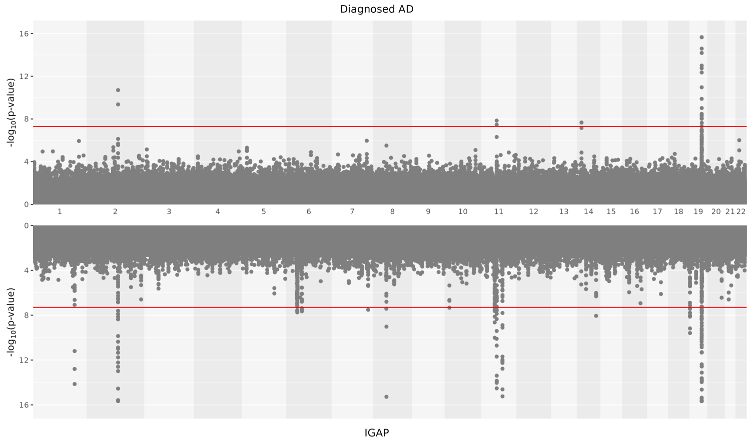

(a)

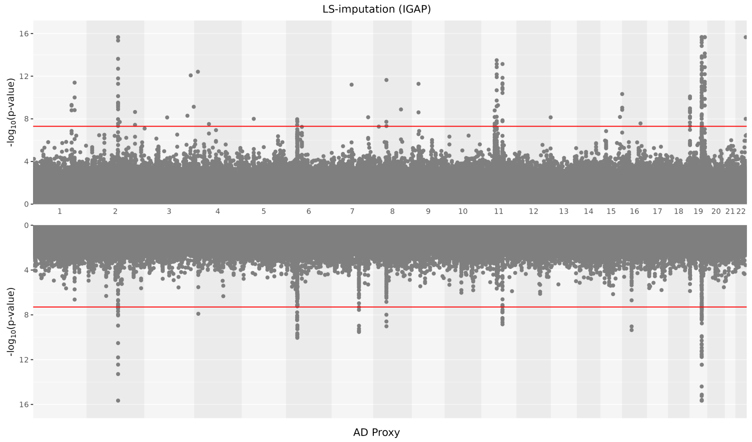

(b)

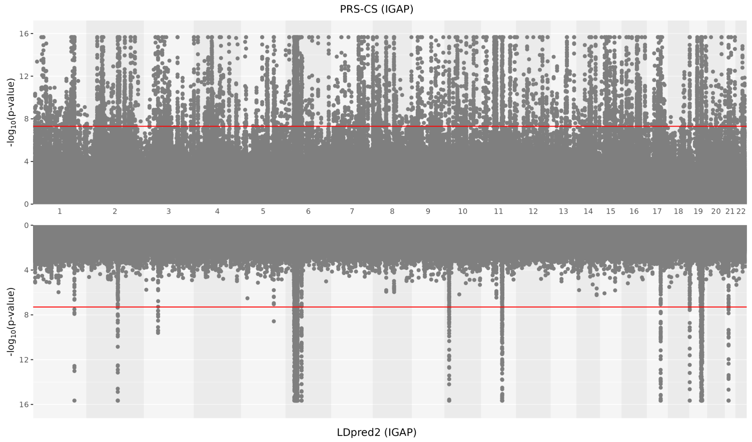

(c)

Figure M: Mirrored Manhattan plots: (a) Diagnosed AD (UKB) vs IGAP; (b) LS-imputation (trained on IGAP) vs AD Proxy (UKB); (c) PRS-CS (trained on IGAP) vs LDpred2 (trained on IGAP). The horizontal red lines correspond to the genome-wide significance level of 5×10^{-8}.

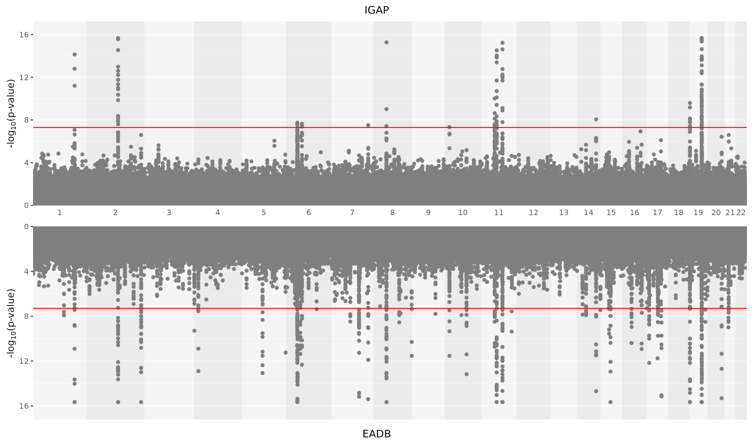

(a)

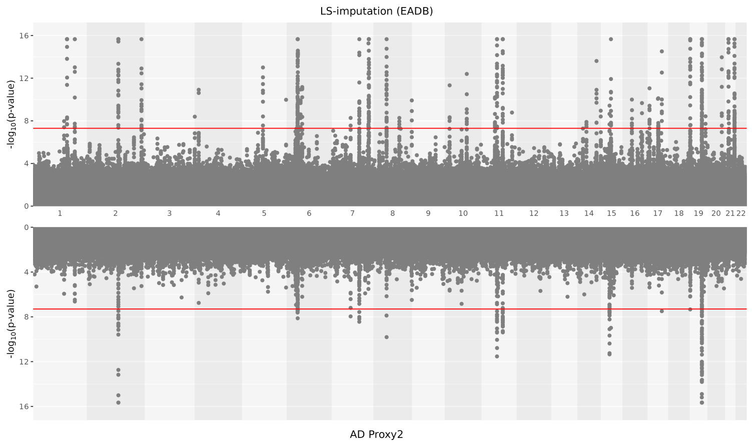

(b)

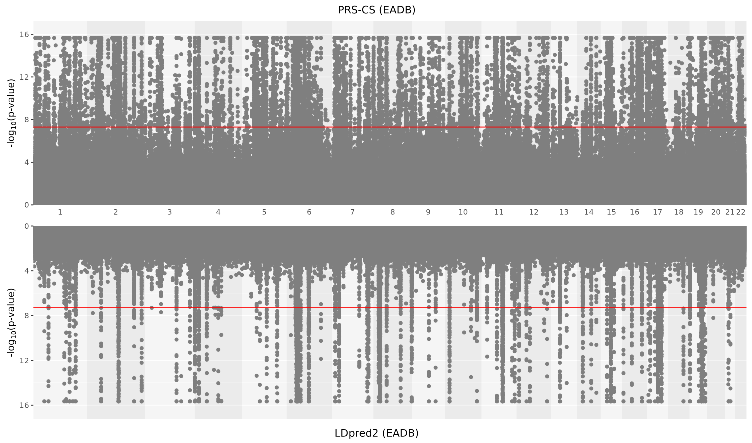

(c)

Figure N: Mirrored Manhattan plots: (a) IGAP vs EADB; (b) LS-imputation (trained on EADB) vs AD Proxy2 (UKB); (c) PRS-CS (trained on EADB) vs LDpred2 (trained on EADB). The horizontal red lines correspond to the genome-wide significance level of 5×10^{-8}.

3 Differently imputed AD traits were very weakly correlated

In this section, we examine the relationships between AD statuses obtained using various imputation methods. The methods considered include AD Proxy, AD Proxy2, LS-imputed AD status, PRS-CS imputed AD status, and LDpred2 imputed AD status. For each pair of methods, we fitted either a linear or logistic regression model, using the AD status from one method as the response and the AD status from the other method as the predictor.

Tables A and B present the R^2 values (for linear regression models) and Nagelkerke's R^2 values (for logistic regression models) obtained from these comparisons. Notably, all imputation methods except AD Proxy result in a continuous trait. When comparing two continuous traits, the R^2 value remains the same regardless of which trait is assigned as the response or predictor. However, for pairs that include the binary AD status imputed by AD Proxy, the R^2 and Nagelkerke's R^2 values vary slightly depending on whether AD Proxy is the response or predictor. These differences, however, are negligible.

We observe that the R^2/Nagelkerke's R^2 values between AD Proxy and AD Proxy2 are significantly higher than those for any other pairs, which is unsurprising as both methods primarily utilize participants' parental information. Similarly, the R^2 value between the two linear imputation methods, PRS-CS and LDpred2, is higher than for other pairs. For all remaining pairs, the low R^2/Nagelkerke's R^2 values indicate that the AD status outcomes imputed by different methods are nearly independent of each other. This suggests that aggregating information from these methods could potentially increase power.

4 Simulation

4.1 Setup

The primary focus of our simulation study is to demonstrate Type I error control when using the LS-imputed trait for training, complementing our real-data analysis. Additionally, we explore the potential for power increase, though only for a simple case, as this is more effectively demonstrated using real data.

We randomly selected 100 genes from the UK Biobank data and used their *cis*-SNPs (100kb upstream and downstream of the TSS and TES) after pruning (removing SNPs with pairwise correlation greater than 0.8). Let $Z_{ij} \in \mathbb{R}^{n \times 1}$ denote the i-th SNP of the j-th gene. We also randomly selected 2000 SNPs across the genome, denoted by the SNP matrix G.

Response	Predictor	p-value	R^2/Nagelkerke's R^2
AD Proxy	LS-imputed AD	$< 2 * 10^{-16}$	0.048
	PRS-CS-imputed AD	$< 2 * 10^{-16}$	0.036
	LDpred2-imputed AD	$< 2 * 10^{-16}$	0.0035
	AD Proxy2	$< 2 * 10^{-16}$	0.950
AD Proxy2	AD Proxy	$< 2 * 10^{-16}$	0.788
	LS-imputed AD	$< 2 * 10^{-16}$	0.022
	PRS-CS-imputed AD	$< 2 * 10^{-16}$	0.0174
	LDpred2-imputed AD	$< 2 * 10^{-16}$	0.00174
LS-imputed AD	AD Proxy	$< 2 * 10^{-16}$	0.0273
	AD Proxy2	$< 2 * 10^{-16}$	0.022
	PRS-CS-imputed AD	$< 2 * 10^{-16}$	0.055
	LDpred2-imputed AD	$< 2 * 10^{-16}$	0.0056
PRS-CS-imputed AD	AD Proxy	$< 2 * 10^{-16}$	0.0205
	AD Proxy2	$< 2 * 10^{-16}$	0.0174
	LS-imputed AD	$< 2 * 10^{-16}$	0.055
	LDpred2-imputed AD	$< 2 * 10^{-16}$	0.244
LDpred2-imputed AD	AD Proxy	$< 2 * 10^{-16}$	0.0019
	AD Proxy2	$< 2 * 10^{-16}$	0.00174
	LS-imputed AD	$< 2 * 10^{-16}$	0.0056
	PRS-CS-imputed AD	$< 2 * 10^{-16}$	0.244

Table A: R^2/Nagelkerke's R^2 obtained from regression models with AD traits imputed using different methods as responses and predictors. EADB GWAS summary statistics were used as the training data for PRS-CS, LDpred2, and LS-imputation.

Response	Predictor	p-value	R^2/Nagelkerke's R^2
AD Proxy	LS-imputed AD	$< 2 * 10^{-16}$	0.00058
	PRS-CS-imputed AD	$< 2 * 10^{-16}$	0.014
	LDpred2-imputed AD	$< 2 * 10^{-16}$	0.0070
	AD Proxy2	$< 2 * 10^{-16}$	0.950
AD Proxy2	AD Proxy	$< 2 * 10^{-16}$	0.788
	LS-imputed AD	$< 2 * 10^{-16}$	0.000328
	PRS-CS-imputed AD	$< 2 * 10^{-16}$	0.0080
	LDpred2-imputed AD	$< 2 * 10^{-16}$	0.00385
LS-imputed AD	AD Proxy	$< 2 * 10^{-16}$	0.000328
	AD Proxy2	$< 2 * 10^{-16}$	0.000328
	PRS-CS-imputed AD	$< 2 * 10^{-16}$	0.0402
	LDpred2-imputed AD	$< 2 * 10^{-16}$	0.0038
PRS-CS-imputed AD	AD Proxy	$< 2 * 10^{-16}$	0.00816
	AD Proxy2	$< 2 * 10^{-16}$	0.0080
	LS-imputed AD	$< 2 * 10^{-16}$	0.0402
	LDpred2-imputed AD	$< 2 * 10^{-16}$	0.266
LDpred2-imputed AD	AD Proxy	$< 2 * 10^{-16}$	0.0039
	AD Proxy2	$< 2 * 10^{-16}$	0.0039
	LS-imputed AD	$< 2 * 10^{-16}$	0.0038
	PRS-CS-imputed AD	$< 2 * 10^{-16}$	0.266

Table B: R^2/Nagelkerke's R^2 from regression models with imputed AD status outcomes obtained using different methods as the responses and predictors. IGAP summary statistics were used as the training data for PRS-CS, LDpred2, and LS-imputation.

Data were generated as follows:

$$U_j \sim N(0, 0.007), \tag{1}$$

$$X_j = \sum_i Z_{ij}\beta_{ij} + U_j + e_j, \quad \beta_{ij} \sim N(0.1, 0.1), \tag{2}$$

$$Y = \sum_j (g(X_j) + U_j) + G\alpha + e_Y, \tag{3}$$

where U_j represents an unobserved confounding variable, and e_j and e_Y are independent normally distributed error terms with mean 0 and variance 0.007. The function $g(\cdot)$ is the causal function. For testing Type I error rate control, we set $g(X) = 0$, and for the power

analysis, we defined

$$g(x) = \begin{cases} -0.5 \cdot x, & \text{if } x \leq -0.5, \\ 2 \cdot x, & \text{if } -0.5 < x \leq 0.5, \\ 0.5 \cdot x, & \text{if } x > 0.5. \end{cases}$$

Note that G is necessary when $g(X) = 0$ to perform LS-imputation. Similar to the real-data analysis for HDL, we created four datasets: $(Z^{GWAS}, G^{GWAS}, X^{GWAS}, Y^{GWAS})$, (Z_1, G_1, X_1, Y_1), (Z_2, G_2, X_2, Y_2), and $(Z_2, G_2, X_2, \tilde{Y}_2)$ with sample sizes $137,162$, $1,000$, $39,000$, and $39,000$, respectively. We used $(Z^{GWAS}, G^{GWAS}, X^{GWAS}, Y^{GWAS})$ to calculate the GWAS summary statistics and obtain the imputed trait $\tilde{Y}_2$. We refer to (Z_1, G_1, X_1, Y_1) as the observed data with sample size n_1, (Z_2, G_2, X_2, Y_2) as the complete data with sample size n_2, and $(Z_2, G_2, X_2, \tilde{Y}_2)$ as the imputed data.

We trained DeLIVR on all three datasets and refer to the models as DeLIVR-Observed, DeLIVR-Complete, and DeLIVR-Imputed, respectively. In addition to the deep learning model, we also trained a standard TWAS model on the observed data, referred to as TWAS-L-Observed. All experiments were repeated 100 times.

4.2 DeLIVR trained with the LS-imputed data controlled the Type I error rate at the nominal level and improved power

Table C presents the Type I error rates and power for each method. All methods successfully controlled the Type I error rate at the nominal level of 0.05. DeLIVR-Imputed demonstrated significantly higher power than DeLIVR-Observed and slightly higher power than TWAS-L-Observed, likely because the $g(X)$ function could be well approximated by a linear function. As expected, DeLIVR-Complete achieved the highest power. These simulation results are consistent with the conclusions from the real-data analysis.

	TWAS-L Observed	DeLIVR Observed	DeLIVR Imputed	DeLIVR Complete
Type I error rate	0.052<br>[0.04 - 0.056]	0.048<br>[0.044 - 0.052]	0.052<br>[0.048 - 0.056]	0.047<br>[0.043 - 0.051]
Power	0.57<br>[0.56 - 0.58]	0.22<br>[0.22 - 0.23]	0.59<br>[0.58 - 0.60]	0.98<br>[0.98 - 0.99]

Table C: Empirical Type I error rates and the power for each method. The numbers in brackets are the 95% confidence interval.

5 TWAS/PWAS analysis with different data QC processes and stage 1 models

Here, we present a different QC process and apply LASSO or Elastic-Net as the stage 1 model. This QC process resulted in substantially more genes passing the stage 1 criterion and being included in the stage 2 analysis.

GTEx gene expression data

We used genotype data and whole blood and brain hippocampus gene expression data from GTEx v8, which includes expression data for 19,626 and 23,648 genes, respectively [1]. We subset the GTEx data to individuals with genetically inferred European ancestry ($n = 558$). To preprocess the data, we regressed out the effects of 68 covariates provided by GTEx and used the standardized residuals as the new gene expression levels. We defined the *cis*-SNPs as SNPs located within a 500k bp window around each coding region. PLINK was used to extract the *cis*-SNPs with the following filters: $--$ MAF 0.05 $--$ geno 0 $--$ hwe $1e-15$.

We applied LASSO and Elastic-Net as the stage 1 models. To assess model fit, we used the out-of-sample R^2 of the predicted values. To obtain the out-of-sample R^2 while tuning hyperparameters with CV, we used a double CV approach: a 3-fold outer CV to calculate the out-of-sample R^2 and a 5-fold inner CV to tune the parameters. Any model with an $R^2 < 0.01$ was discarded. If both LASSO and Elastic-Net achieved $R^2 \geq 0.01$, the model with the higher R^2 was selected. A total of 8,516 and 7,339 genes had a stage 1 $R^2 \geq 0.01$ and were included in the stage 2 analysis for the whole blood and brain hippocampus tissues, respectively.

UKB protein expression data

The PLINK filters were identical to those used for the GTEx data. The covariates adjusted for were age, sex, age^2, sex$*$age, sex$*$age^2, and the top 20 genetic PCs. The stage 1 models and selection criteria were also the same. A total of 1,250 proteins had a stage 1 $R^2 \geq 0.01$ and were included in the stage 2 PWAS analysis.

5.0.1 Stage 2 datasets

For the ADSP data, the PLINK filters remained the same, while for the UK Biobank data, we used $--$ hwe $1e-5$ 1000, as recommended by PLINK.

5.1 DeLIVR uniquely identified genes/proteins related to AD using imputed AD status

The p-values obtained using whole blood gene expression data are provided in Tables D and E. Fig P illustrates the UpSet plot and Q-Q plot for these results. Using the more stringent Bonferroni cutoff, DeLIVR trained on imputed traits identified one unique gene. In contrast, a relaxed cutoff revealed substantially more unique genes compared to training on the observed trait. Among these genes, *GNAZ*, for instance, has been previously shown to be associated with AD [2]. The Cauchy combination test identified the highest number of genes, tied with the results obtained using "LS-imp IGAP." However, aggregating results across different imputed traits produced a distinct set of genes compared to "LS-imp IGAP," leveraging complementary information from multiple imputations. Although models trained on imputed traits identified more genes than those trained on the observed trait, the Q-Q plot confirms that the p-values were well-calibrated, demonstrating the robustness of the approach.

Tables F and G, along with Fig Q, present the results obtained using brain hippocampus tissue. The overall conclusions are consistent with those derived from the whole blood analysis, with one notable difference: the Cauchy combination test identified substantially more genes than any single imputed trait alone. This improvement is likely due to the greater distinctiveness of the gene sets identified by each imputed trait.

Tables H and I, as well as Fig R, present PWAS results. Similar to the TWAS analysis, DeLIVR uniquely identified proteins related to AD. For example, *MMP13* has been reported to be associated with AD pathogenesis and might represent a potential drug target for AD [3]. Using the relaxed significance cutoff ($1e-3$), TWAS-L identified the largest number of proteins, potentially due to the relatively linear association between protein expression and AD. The Q-Q plot shows that the distributions of p-values for all models are similar and well-calibrated.

6 TWAS/PWAS results with significance cutoff 1×10^{-3}

In this section, we present the TWAS (Table J) and PWAS (Table K) p-values using a significance cutoff of 1×10^{-3}. The QC process and stage 1 model are consistent with those described in Section 2.4 of the main text.

Model				DeLIVR					TWAS-L	TWAS-LQ
Training Data				ADSP	LS-imp IGAP	LS-imp EADB	AD Proxy	Combined	ADSP	ADSP
Gene	chr	TSS	TES							
MS4A4A	11	60280540	60280541	1.11e-02	**3.72e-07**	5.18e-04	**4.06e-07**	**5.83e-07**	**3.43e-07**	**1.68e-06**
MS4A6A	11	60184665	60184666	5.73e-04	3.35e-04	4.73e-04	5.57e-04	4.35e-04	4.17e-06	NA
RP11-395L14.18	2	113607907	113607908	2.12e-01	**3.04e-07**	2.68e-04	2.11e-02	**9.11e-07**	7.25e-03	NA
RP11-672L10.6	18	813273	813274	**4.09e-07**	2.72e-04	**1.43e-06**	**1.05e-10**	**3.14e-10**	1.54e-03	6.66e-03
SCFD1	14	30622111	30622112	**2.23e-09**	3.37e-04	7.41e-04	2.15e-02	6.88e-04	9.17e-01	1.30e-01
WASH2P	2	113588549	113588550	**5.86e-08**	1.35e-05	9.88e-01	4.96e-03	4.04e-05	4.06e-04	1.38e-03

Table D: TWAS results using whole blood gene expression. The table shows the p-values of genes identified by at least one method. The Bonferroni cutoff is 5.9×10^{-6}. The top row lists the models evaluated, while the second row indicates the training datasets used. "Combined" refers to the combined p-values from "LS-imp IGAP," "LS-imp EADB," and "AD Proxy." All models were tested on the ADSP data. p-values smaller than the Bonferroni cutoff are highlighted in bold.

References

[1] François Aguet et al. "The GTEx Consortium atlas of genetic regulatory effects across human tissues." In: *Science* 369.6509 (2020), pp. 1318–1330.

[2] S Akila Parvathy Dharshini, YH Taguchi, and M Michael Gromiha. "Exploring the selective vulnerability in Alzheimer disease using tissue specific variant analysis." In: *Genomics* 111.4 (2019), pp. 936–949.

[3] Jean-Michel Paumier and Gopal Thinakaran. "Matrix metalloproteinase 13, a new target for therapy in Alzheimer's disease." In: *Genes & Diseases* 6.1 (2019), p. 1.

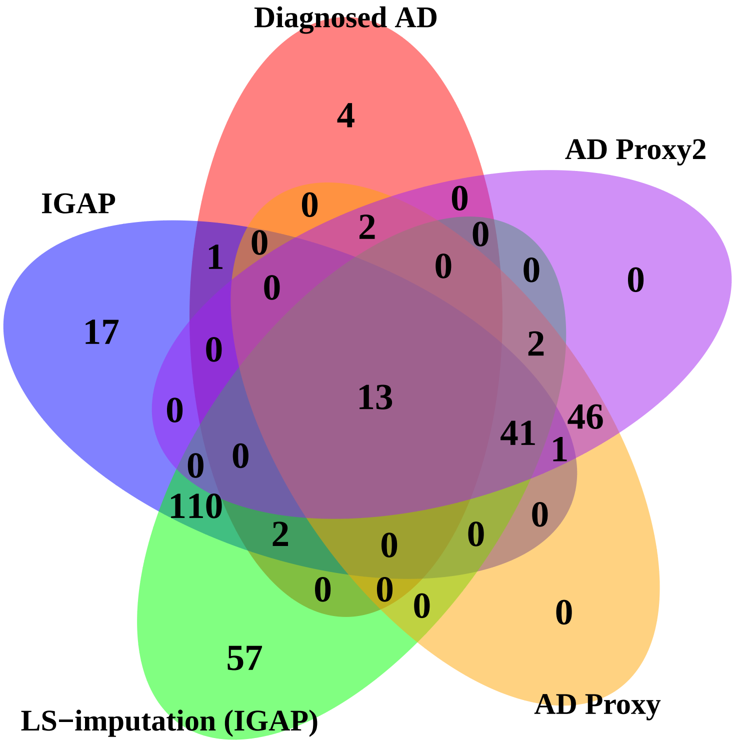

(a)

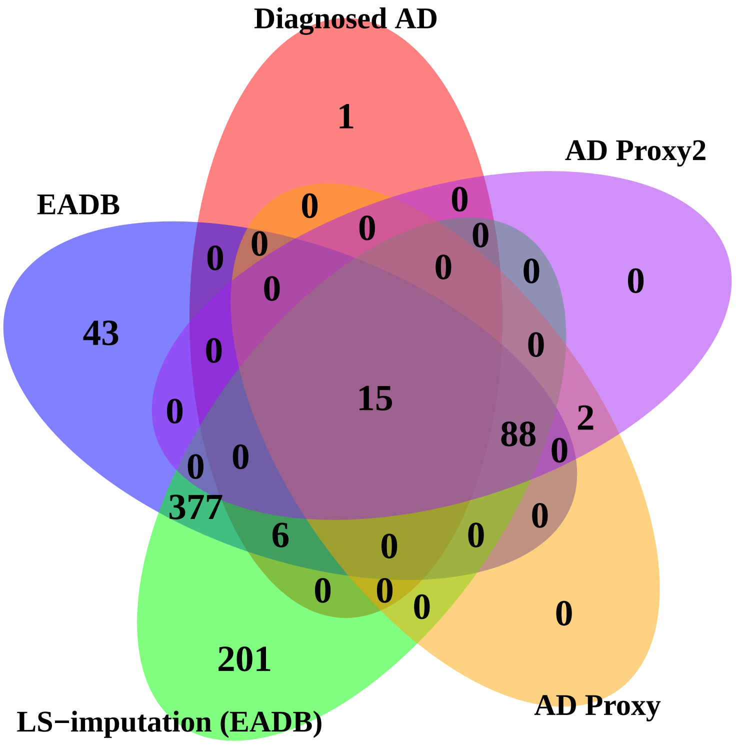

(b)

Figure O: Venn diagrams of the significant SNPs (cutoff $5e-8$) identified by GWAS analyzed on different traits. (a) IGAP; (b) EADB.

Model				DeLIVR					TWAS-L	TWAS-LQ
Training Data				ADSP	LS-imp IGAP	LS-imp EADB	AD Proxy	Combined	ADSP	ADSP
Gene	chr	TSS	TES							
RP11-672L10.6	18	813273	813274	4.09e-07	2.72e-04	1.43e-06	1.05e-10	3.14e-10	1.54e-03	6.66e-03
MS4A4A	11	60280540	60280541	1.11e-02	3.72e-07	5.18e-04	4.06e-07	5.83e-07	3.43e-07	1.68e-06
RP11-395L14.18	2	113607907	113607908	2.12e-01	**3.04e-07**	2.68e-04	2.11e-02	**9.11e-07**	7.25e-03	NA
WASH2P	2	113588549	113588550	5.86e-08	1.35e-05	9.88e-01	4.96e-03	4.04e-05	4.06e-04	1.38e-03
KREMEN1	22	29073077	29073078	8.12e-03	1.01e-03	1.56e-05	1.71e-04	4.22e-05	1.73e-04	NA
P2RX7	12	121132818	121132819	1.07e-01	2.73e-04	2.23e-02	4.83e-05	1.23e-04	6.87e-03	2.25e-02
TRIM35	8	27311318	27311319	3.04e-01	4.77e-01	1.82e-02	**6.04e-05**	1.81e-04	8.34e-02	NA
TNFSF13	17	7558291	7558292	9.15e-02	1.88e-03	**6.87e-05**	1.76e-02	1.98e-04	5.03e-02	1.40e-01
RP11-689B22.2	12	108628686	108628687	2.56e-01	4.79e-02	7.60e-05	6.61e-04	2.04e-04	3.04e-03	9.57e-04
CPD	17	30378904	30378905	8.31e-01	1.01e-04	3.17e-04	7.87e-04	2.09e-04	4.37e-04	NA
MGMT	10	129467183	129467184	1.64e-02	**3.09e-04**	**1.25e-04**	5.47e-02	**2.67e-04**	3.01e-02	1.06e-02
KIAA1324L	7	87059698	87059699	7.27e-01	**1.23e-04**	3.72e-02	5.97e-04	**3.05e-04**	1.20e-01	NA
ISCA2	14	74493719	74493720	2.27e-02	**3.89e-04**	**3.75e-04**	**3.59e-04**	**3.74e-04**	2.13e-02	NA
MS4A6A	11	60184665	60184666	5.73e-04	3.35e-04	4.73e-04	5.57e-04	4.35e-04	4.17e-06	NA
GNAZ	22	23070360	23070361	4.12e-02	**7.21e-04**	**2.98e-04**	**5.20e-04**	**4.50e-04**	1.13e-02	NA
TMBIM6	12	49707724	49707725	6.34e-01	**1.56e-04**	7.34e-01	3.83e-01	**4.69e-04**	3.65e-01	4.16e-01
AFDN	6	167826921	167826922	2.65e-03	1.20e-02	7.45e-02	1.64e-04	4.83e-04	6.02e-01	6.92e-01
ZFYVE28	4	2418662	2418663	1.34e-02	5.74e-04	8.37e-04	3.52e-04	5.19e-04	7.35e-02	NA
AIFM2	10	70132933	70132934	1.61e-02	3.06e-04	7.23e-04	1.91e-03	5.80e-04	4.40e-04	1.62e-03
SCFD1	14	30622111	30622112	2.23e-09	3.37e-04	7.41e-04	2.15e-02	6.88e-04	9.17e-01	1.30e-01
DSE	6	116254172	116254173	1.01e-02	6.60e-01	**2.49e-04**	9.68e-02	**7.45e-04**	8.70e-01	7.77e-01
RP11-299G20.5	15	101301647	101301648	4.10e-02	2.48e-01	3.11e-04	8.93e-03	9.01e-04	7.68e-03	NA
BLZF1	1	169367969	169367970	8.63e-02	4.72e-01	3.31e-01	**3.17e-04**	**9.49e-04**	2.12e-01	3.64e-02
ZNF304	19	57351359	57351360	1.20e-01	3.73e-04	2.25e-01	4.92e-03	1.04e-03	1.47e-01	1.64e-01
SYT11	1	155859508	155859509	8.12e-01	1.46e-03	5.36e-04	3.55e-03	1.06e-03	1.15e-03	NA
CTD-2020K17.4	17	45248835	45248836	2.80e-02	7.02e-04	1.41e-02	1.18e-03	1.28e-03	1.03e-03	NA
VDR	12	47943047	47943048	9.28e-01	**4.50e-04**	4.24e-02	1.68e-02	1.30e-03	4.80e-01	3.91e-01
SUGT1	13	52652708	52652709	8.00e-01	3.04e-01	**4.65e-04**	8.62e-03	1.32e-03	2.38e-01	NA
ZNF765	19	53389792	53389793	1.27e-03	7.47e-04	3.48e-03	1.01e-02	1.74e-03	7.24e-02	1.26e-01
GPD2	2	156435289	156435290	2.86e-01	2.44e-02	3.95e-02	6.44e-04	1.85e-03	3.26e-03	5.73e-03
RP3-395M20.8	1	2557030	2557031	7.69e-02	5.32e-03	1.97e-02	7.39e-04	1.89e-03	1.41e-01	1.80e-01
RP11-536K7.3	10	5945899	5945900	4.42e-02	5.16e-03	**8.34e-04**	5.63e-03	1.91e-03	7.63e-01	NA
BRI3	7	98252378	98252379	2.45e-01	**7.87e-04**	4.71e-02	9.44e-03	2.15e-03	5.12e-02	NA
CA3-AS1	8	85464914	85464915	3.19e-02	8.15e-04	4.69e-02	1.12e-02	2.24e-03	3.09e-02	2.48e-02
SPOCK2	10	72089031	72089032	1.36e-02	8.52e-01	9.22e-03	8.97e-04	2.47e-03	2.04e-02	5.20e-02
GLS	2	190895151	190895152	1.42e-01	8.86e-04	3.44e-02	1.77e-02	2.47e-03	3.19e-02	NA
TRMT12	8	124450819	124450820	9.90e-02	6.25e-01	1.15e-01	8.98e-04	2.68e-03	1.70e-03	6.16e-03
RP11-212I21.4	16	55538199	55538200	6.51e-01	2.31e-02	3.65e-02	9.52e-04	2.68e-03	4.29e-01	7.12e-01
NDUFAF1	15	41402518	41402519	3.08e-01	7.41e-02	9.88e-04	4.46e-02	2.86e-03	9.79e-01	1.57e-02
PRSS57	19	695497	695498	1.25e-01	8.66e-01	5.02e-01	1.48e-03	4.48e-03	8.38e-01	8.84e-04
YME1L1	10	27154822	27154823	3.69e-02	1.05e-01	8.29e-02	1.70e-03	4.91e-03	7.74e-04	NA
GUSBP5	4	143559471	143559472	3.14e-02	2.06e-02	2.50e-03	7.91e-03	5.22e-03	7.21e-04	NA
MASTL	10	27155266	27155267	5.19e-01	5.14e-03	5.33e-03	5.61e-02	7.50e-03	4.88e-04	1.58e-04
KLC3	19	45333433	45333434	1.51e-04	5.57e-03	3.76e-01	4.72e-03	7.64e-03	1.43e-03	NA
IRGQ	19	43596134	43596135	2.70e-01	1.13e-02	6.52e-03	7.62e-01	1.26e-02	5.62e-04	9.51e-04
VN1R1	19	57457141	57457142	7.81e-04	5.66e-03	4.70e-02	3.90e-02	1.34e-02	1.24e-01	3.04e-01
KAT5	11	65711995	65711996	3.77e-01	9.43e-03	3.54e-02	1.77e-01	2.15e-02	8.88e-04	3.77e-03
GPS2	17	7315563	7315564	1.91e-05	1.72e-02	5.13e-02	3.20e-02	2.76e-02	5.04e-02	NA
C17orf107	17	4899417	4899418	5.56e-01	8.57e-02	3.02e-02	2.49e-02	3.53e-02	1.72e-02	8.28e-05
MICAL3	22	18024558	18024559	4.38e-04	4.74e-01	1.68e-02	2.23e-01	4.68e-02	2.70e-01	NA
ELL	19	18522126	18522127	1.41e-01	1.15e-01	9.99e-02	2.21e-02	4.72e-02	3.38e-04	9.33e-04
CLDN10	13	95433603	95433604	3.68e-01	9.75e-02	3.67e-02	7.98e-02	6.01e-02	5.24e-04	2.34e-03
SLC35C1	11	45804071	45804072	3.50e-04	1.39e-01	2.25e-01	1.24e-01	1.53e-01	3.82e-01	NA
GOLGA8R	15	30414161	30414162	2.29e-04	5.58e-01	8.63e-01	4.33e-02	1.74e-01	3.43e-02	NA
AGAP9	10	47523637	47523638	6.74e-01	2.07e-01	2.99e-01	2.96e-01	2.62e-01	3.85e-01	5.59e-04
KLF11	2	10042848	10042849	6.50e-04	5.89e-01	7.29e-01	5.73e-01	6.39e-01	6.19e-01	8.36e-01
BMS1P2	10	47551953	47551954	3.35e-02	8.93e-01	8.21e-01	8.32e-01	8.56e-01	7.17e-02	1.52e-04
MRPL18	6	159789811	159789812	6.48e-02	9.85e-01	8.95e-01	1.97e-01	9.58e-01	6.41e-02	4.89e-04
KCNMA1	10	77638368	77638369	2.11e-01	**7.43e-04**	7.85e-01	1.00e+00	9.99e-01	1.46e-01	3.30e-01

Table E: TWAS results using whole blood gene expression. The table shows the p-values of genes identified by at least one method. The significance cutoff is 1×10^{-3}. The top row lists the models evaluated, while the second row indicates the training datasets used. "Combined" refers to the combined p-values from "LS-imp IGAP," "LS-imp EADB," and "AD Proxy." All models were tested on the ADSP data. p-values from the DeLIVR method

Model				DeLIVR					TWAS-L	TWAS-LQ
Training Data				ADSP	LS-imp IGAP	LS-imp EADB	AD Proxy	LS-imp Combined	ADSP	ADSP
Gene	chr	TSS	TES							
PROC	2	127418426	127418427	9.15e-02	3.81e-01	7.40e-04	7.64e-02	2.20e-03	2.73e-04	**1.25e-05**
WASH2P	2	113588549	113588550	6.84e-03	2.88e-04	1.92e-03	**6.66e-06**	1.95e-05	2.17e-04	8.75e-04

Table F: TWAS results using brain hippocampus gene expression. The table shows the p-values of genes identified by at least one method. The Bonferroni cutoff is 6.8×10^{-6}. The top row lists the models evaluated, while the second row indicates the training datasets used. "Combined" refers to the combined p-values from "LS-imp IGAP," "LS-imp EADB," and "AD Proxy." All models were tested on the ADSP data. p-values smaller than the Bonferroni cutoff are highlighted in bold.

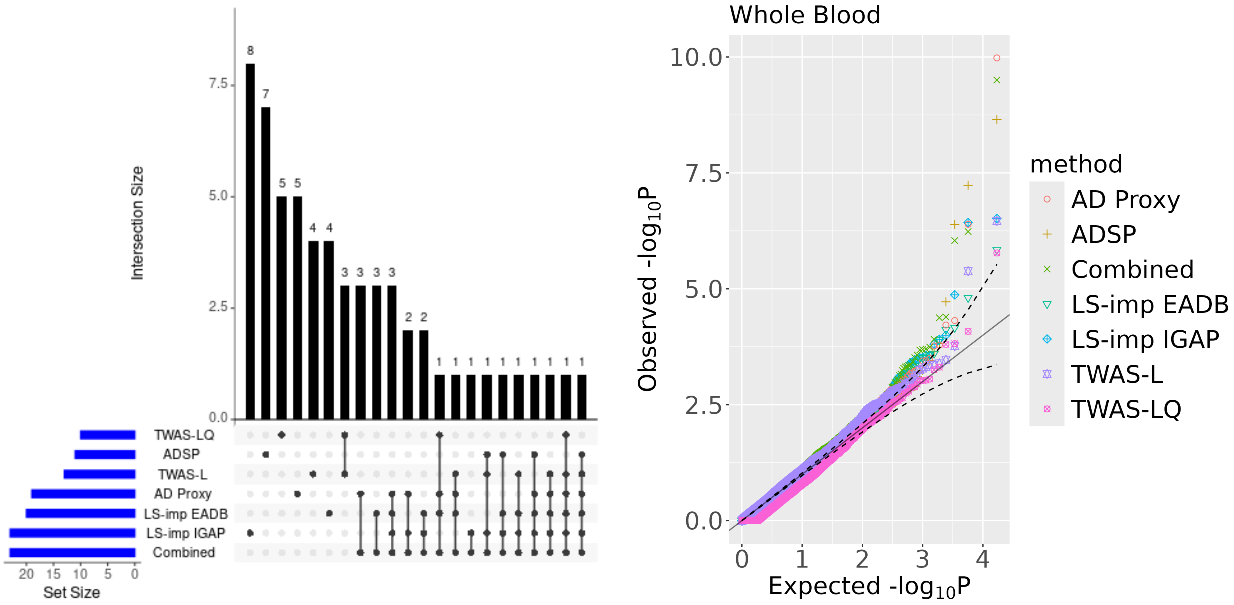

(a) UpSet plot.

(b) Q-Q plot.

Figure P: Side-by-side UpSet plot and Q-Q plot of all models for TWAS using whole blood gene expression. "ADSP" refers to the DeLIVR model trained on the ADSP data (observed AD status). "TWAS-L" and "TWAS-LQ" refer to the standard TWAS model and the parametric TWAS-LQ model trained on the ADSP data, respectively. "LS-imp EADB" and "LS-imp IGAP" refer to the DeLIVR model trained on the LS-imputed AD status using EADB and IGAP as the GWAS data, respectively. "AD Proxy" refers to the DeLIVR model trained on the proxy AD status. "Combined" refers to the results of the Cauchy combination test, which combines the p-values of "LS-imp EADB," "LS-imp IGAP," and "AD Proxy."

Model				DeLIVR					TWAS-L	TWAS-LQ
Training Data				ADSP	LS-imp IGAP	LS-imp EADB	AD Proxy	Combined	ADSP	ADSP
Gene	chr	TSS	TES							
WASH2P	2	113588549	113588550	6.84e-03	2.88e-04	1.92e-03	6.66e-06	1.95e-05	2.17e-04	8.75e-04
CR1	1	207496146	207496147	1.69e-02	2.96e-01	7.09e-04	1.24e-05	3.65e-05	1.22e-05	NA
STAM	10	17644124	17644125	1.27e-01	4.34e-02	**4.60e-05**	6.30e-02	1.38e-04	2.22e-02	NA
FOXJ2	12	8032702	8032703	7.88e-02	2.33e-04	2.94e-04	8.96e-05	1.59e-04	1.46e-03	NA
C12orf54	12	48482502	48482503	8.51e-01	8.42e-01	9.41e-02	**5.34e-05**	1.60e-04	2.62e-02	NA
EIF5A	17	7306998	7306999	3.57e-01	1.13e-01	6.53e-01	**5.74e-05**	1.72e-04	2.91e-01	5.71e-01
CASZ1	1	10796649	10796650	9.47e-02	7.25e-01	2.59e-01	**6.11e-05**	1.83e-04	5.50e-02	NA
OMA1	1	58546801	58546802	6.20e-03	3.96e-04	1.60e-04	3.01e-04	2.48e-04	2.11e-03	NA
ZNF610	19	52336244	52336245	4.16e-01	1.35e-04	**8.34e-04**	6.87e-02	**3.48e-04**	7.26e-01	NA
LRRC37A	17	46292732	46292733	2.63e-01	1.35e-04	2.17e-03	2.11e-02	3.78e-04	2.48e-03	NA
SRGAP3-AS4	3	9256758	9256759	2.95e-01	5.86e-01	**1.40e-04**	5.32e-01	**4.19e-04**	2.30e-01	NA
RP11-395L14.18	2	113607907	113607908	5.34e-04	5.15e-01	1.45e-02	1.68e-04	4.99e-04	4.96e-04	NA
SLC47A2	17	19718978	19718979	1.80e-01	2.71e-02	1.85e-04	6.92e-02	5.50e-04	3.80e-03	NA
LRRC37A6P	10	27259454	27259455	7.48e-02	7.26e-04	6.87e-04	5.90e-04	6.63e-04	5.45e-04	1.79e-03
CTB-171A8.1	19	44718758	44718759	2.48e-02	6.90e-04	3.42e-04	4.47e-02	6.82e-04	1.16e-05	NA
FUT2	19	48695970	48695971	1.74e-01	2.73e-03	8.35e-02	2.64e-04	7.21e-04	2.18e-04	NA
FBXO24	7	100583981	100583982	3.52e-02	**2.73e-04**	3.82e-03	2.64e-02	**7.57e-04**	4.82e-02	1.41e-01
RP11-770J1.4	11	118435205	118435206	2.90e-02	6.07e-04	5.71e-04	1.88e-03	7.63e-04	4.68e-04	1.08e-03
MX2	21	41361942	41361943	8.96e-03	6.43e-03	4.34e-01	2.82e-04	8.10e-04	2.04e-03	5.80e-03
PGM5P4	2	113541936	113541937	1.80e-02	1.55e-03	3.85e-04	7.80e-03	8.89e-04	1.14e-03	NA
SLC7A9	19	32869765	32869766	6.92e-01	4.06e-02	2.12e-03	**4.54e-04**	1.11e-03	1.76e-01	NA
ASAH1	8	18084997	18084998	2.32e-02	3.52e-03	6.06e-04	2.22e-03	1.26e-03	1.77e-02	5.66e-02
C4A	6	31982023	31982024	8.70e-01	9.26e-01	1.13e-01	4.36e-04	1.31e-03	3.99e-02	NA
WDR6	3	49007154	49007155	3.75e-03	**4.71e-04**	2.62e-02	1.02e-02	1.33e-03	4.21e-01	2.58e-01
NPIPA5	16	15381046	15381047	7.71e-01	5.00e-03	8.97e-01	5.30e-04	1.45e-03	3.60e-01	NA
NOTCH4	6	32224066	32224067	1.00e-01	2.19e-02	2.09e-03	6.55e-04	1.46e-03	2.62e-02	NA
PRODH	22	18936409	18936410	2.78e-01	**4.97e-04**	2.54e-02	9.11e-01	1.47e-03	9.55e-01	3.94e-01
DDX11L2	2	113601260	113601261	1.09e-01	1.01e-03	2.03e-02	1.33e-03	1.67e-03	2.94e-04	NA
PROC	2	127418426	127418427	9.15e-02	3.81e-01	7.40e-04	7.64e-02	2.20e-03	2.73e-04	1.25e-05
MASTL	10	27155266	27155267	8.45e-02	5.28e-03	9.15e-04	1.70e-02	2.24e-03	3.42e-04	7.64e-04
ZNF154	19	57709193	57709194	5.98e-01	**8.01e-04**	7.37e-01	7.44e-01	2.41e-03	8.27e-01	NA
CYB561	17	63446377	63446378	4.62e-02	3.77e-03	1.77e-03	3.28e-03	2.64e-03	8.86e-04	3.66e-03
CA3-AS1	8	85464914	85464915	1.08e-01	9.29e-02	9.12e-02	9.45e-04	2.78e-03	9.91e-03	NA
MTRNR2L3	20	57359979	57359980	1.11e-01	3.97e-03	2.16e-03	1.17e-02	3.75e-03	3.91e-04	NA
GDPD3	16	30113855	30113856	8.73e-04	2.73e-03	3.10e-01	8.05e-03	6.09e-03	9.24e-01	7.31e-01
ZNF296	19	45076508	45076509	2.89e-01	5.57e-03	4.04e-02	1.40e-01	1.42e-02	4.41e-05	8.83e-05
CHRFAM7A	15	30393848	30393849	7.74e-02	1.77e-02	6.78e-01	6.47e-03	1.44e-02	2.41e-03	9.85e-04
CTD-2336O2.3	8	1760446	1760447	1.61e-01	1.01e-02	2.89e-01	1.10e-01	2.72e-02	5.87e-04	NA
KIRREL3	11	127001164	127001165	2.84e-04	8.85e-02	1.31e-02	5.49e-02	2.84e-02	2.98e-02	NA
TANGO2	22	20017013	20017014	2.90e-01	9.87e-02	1.54e-01	1.23e-02	3.07e-02	2.20e-02	1.44e-04
ARFGAP3	22	42858105	42858106	3.89e-04	9.48e-02	3.22e-02	2.18e-02	3.43e-02	1.52e-01	NA
C15orf57	15	40565056	40565057	2.10e-02	4.87e-02	7.20e-02	1.92e-02	3.47e-02	6.00e-03	6.90e-04
RPL23AP7	2	113627089	113627090	1.62e-01	5.07e-02	2.10e-02	4.35e-01	4.40e-02	8.50e-04	NA
CTC-329D1.2	5	140401366	140401367	3.40e-04	6.42e-01	2.03e-02	4.99e-02	4.41e-02	1.03e-01	8.08e-02
RP11-472B18.1	4	39480254	39480255	3.02e-02	6.96e-02	1.37e-01	2.80e-02	5.25e-02	2.05e-04	9.71e-04
CPXM1	20	2800636	2800637	1.56e-04	8.31e-02	3.58e-02	7.44e-02	5.63e-02	4.61e-02	9.54e-02
AC142472.6	17	45148469	45148470	1.64e-02	4.29e-01	2.40e-02	1.34e-01	6.02e-02	4.26e-04	2.00e-03
LINC01567	16	24671061	24671062	7.55e-04	3.37e-02	4.27e-01	4.54e-01	9.47e-02	6.54e-01	4.26e-01
IGF2R	6	159969098	159969099	1.46e-01	8.90e-02	1.99e-01	3.08e-01	1.58e-01	4.63e-04	1.02e-03
LRRC37A2	17	46511510	46511511	7.74e-01	3.45e-01	2.41e-01	7.90e-02	1.58e-01	3.12e-02	1.29e-04
ZNF488	10	47384272	47384273	1.75e-02	4.02e-01	1.79e-01	1.32e-01	1.98e-01	8.12e-04	3.62e-03
CPLX3	15	74826546	74826547	4.76e-04	5.67e-01	7.80e-02	3.95e-01	2.00e-01	4.40e-01	NA
AL163953.3	14	53169053	53169054	4.93e-04	6.60e-01	1.45e-01	8.09e-01	4.97e-01	1.88e-01	NA
ATP6V1G1P2	8	47194346	47194347	5.49e-04	8.95e-01	9.38e-01	7.30e-01	8.96e-01	2.99e-01	NA

Table G: TWAS results using brain hippocampus gene expression. The table shows the p-values of genes identified by at least one method. The significance cutoff is 1×10^{-3}. The top row lists the models evaluated, while the second row indicates the training datasets used. "Combined" refers to the combined p-values from "LS-imp IGAP," "LS-imp EADB," and "AD Proxy." All models were tested on the ADSP data. p-values from the DeLIVR method trained on imputed traits that are significantly smaller than those from models trained on observed traits are highlighted.

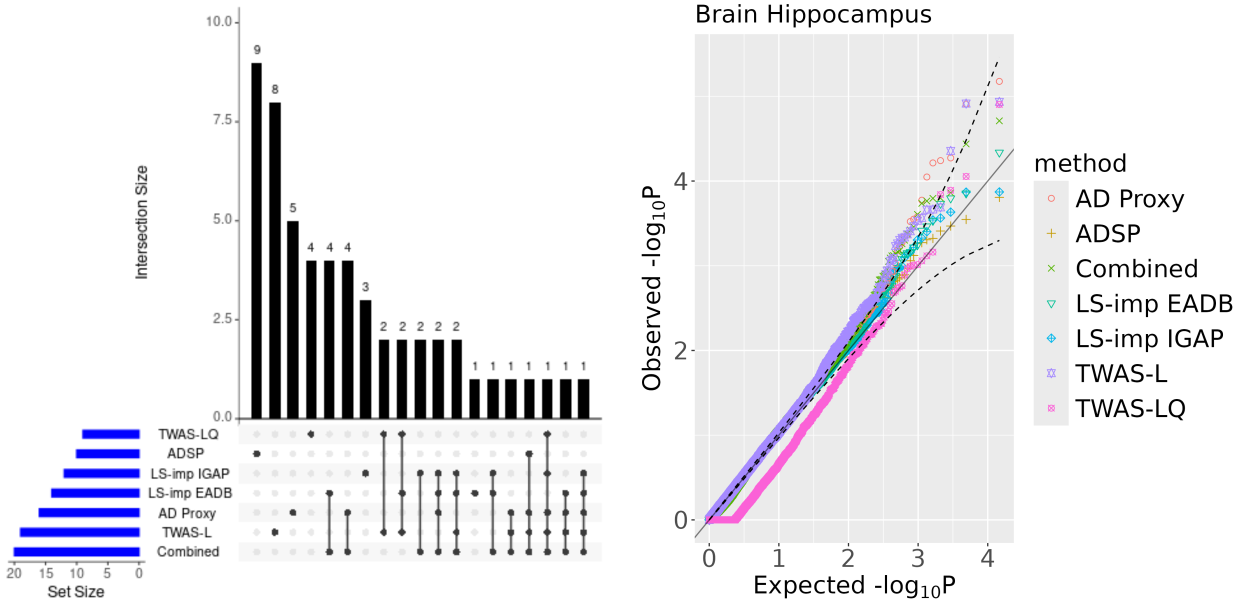

(a) UpSet plot.

(b) Q-Q plot.

Figure Q: Side-by-side UpSet plot and Q-Q plot of all models for TWAS using brain hippocampus gene expression. "ADSP" refers to the DeLIVR model trained on the ADSP data (observed AD status). "TWAS-L" and "TWAS-LQ" refer to the standard TWAS model and the parametric TWAS-LQ model trained on the ADSP data, respectively. "LS-imp EADB" and "LS-imp IGAP" refer to the DeLIVR model trained on the LS-imputed AD status using EADB and IGAP as the GWAS data, respectively. "AD Proxy" refers to the DeLIVR model trained on the proxy AD status. "Combined" refers to the results of the Cauchy combination test, which combines the p-values of "LS-imp EADB," "LS-imp IGAP," and "AD Proxy."

Model					DeLIVR					TWAS-L	TWAS-LQ
Training Data					ADSP	LS-imp IGAP	LS-imp EADB	AD Proxy	Combined	ADSP	ADSP
Gene	Protein	chr	start	end							
APOE	Apolipoprotein E	19	45409011	45412650	**0.00e+00**	**0.00e+00**	**0.00e+00**	**0.00e+00**	**0.00e+00**	**5.40e-112**	**1.84e-121**
CR1	Complement receptor type 1	1	207669492	207813992	1.15e-03	**3.15e-05**	**1.91e-06**	2.09e-02	**5.39e-06**	**3.72e-08**	**2.64e-07**
ERBB3	Receptor tyrosine-protein kinase erbB-3	12	56473641	56497289	5.57e-02	8.57e-03	4.61e-02	4.34e-02	1.86e-02	**4.60e-06**	**2.33e-05**
LCN15	Lipocalin-15	9	139654086	139660707	7.69e-03	6.59e-02	1.88e-01	3.58e-02	6.23e-02	2.33e-03	**4.06e-06**
PILRA	Paired immunoglobulin-like type 2 receptor alpha	7	99965153	99997719	2.08e-03	1.84e-02	3.85e-02	2.73e-02	2.57e-02	7.59e-04	**3.38e-05**

Table H: PWAS p-values for proteins identified by at least one method. The Bonferroni cutoff is 4×10^{-5}. The top row lists the models evaluated, while the second row indicates the training datasets used. "Combined" refers to the combined p-values from "LS-imp IGAP," "LS-imp EADB," and "AD Proxy." All models were tested on the ADSP data. p-values smaller than the Bonferroni cutoff are highlighted in bold.

Model					DeLIVR					TWAS-L	TWAS-LQ
Training Data					ADSP	LS-imp IGAP	LS-imp EADB	AD Proxy	Combined	ADSP	ADSP
Gene	Protein	chr	start	end							
APOE	Apolipoprotein E	19	45409011	45412650	0.00e+00	0.00e+00	0.00e+00	0.00e+00	0.00e+00	5.40e-112	1.84e-121
CR1	Complement receptor type 1	1	207669492	207813992	1.15e-03	3.15e-05	1.91e-06	2.09e-02	5.39e-06	3.72e-08	2.64e-07
SUMF2	Inactive C-alpha-formylglycine-generating enzyme 2	7	56131695	56148363	1.33e-03	5.03e-05	1.22e-04	6.14e-03	1.06e-04	8.90e-03	1.14e-02
CD276	CD276 antigen	15	73976307	74006859	4.83e-02	2.89e-01	**1.56e-04**	1.17e-03	**4.13e-04**	6.33e-01	2.91e-01
PILRB	Paired immunoglobulin-like type 2 receptor beta	7	99933737	99965356	6.69e-02	2.92e-04	5.89e-04	9.52e-02	5.85e-04	8.21e-04	7.30e-04
APOC1	Apolipoprotein C-I	19	45417504	45422606	1.38e-03	3.46e-04	4.98e-02	2.52e-03	9.07e-04	5.10e-04	1.96e-03
CLPS	Colipase	6	35762759	35765088	6.14e-01	1.98e-03	3.03e-02	**5.02e-04**	1.19e-03	1.61e-01	3.12e-01
CTSH	Pro-cathepsin H	15	79213400	79241916	2.39e-02	4.10e-04	1.20e-02	5.88e-01	1.19e-03	1.14e-02	2.07e-03
VSIG10L	V-set and immunoglobulin domain-containing protein 10-like	19	51834790	51845378	9.62e-03	1.60e-03	9.21e-04	1.70e-03	1.31e-03	4.29e-04	1.17e-03
KAZALD1	Kazal-type serine protease inhibitor domain-containing protein 1	10	102821598	102827888	5.09e-01	5.66e-02	5.50e-01	**4.43e-04**	1.32e-03	4.15e-01	7.14e-01
CEACAM19	Carcinoembryonic antigen-related cell adhesion molecule 19	19	45165545	45187631	6.83e-02	7.06e-01	3.80e-03	5.02e-04	1.33e-03	8.02e-04	NA
MMP13	Collagenase 3	11	102813724	102826463	3.51e-01	4.43e-02	2.96e-02	**5.90e-04**	1.71e-03	7.09e-02	1.73e-01
APOL1	Apolipoprotein L1	22	36649056	36663576	2.84e-02	9.10e-04	8.47e-01	2.73e-03	2.06e-03	2.83e-03	1.00e-02
PINLYP	phospholipase A2 inhibitor and Ly6/PLAUR domain-containing protein	19	44080952	44088116	9.47e-02	3.25e-03	2.68e-01	1.88e-02	8.25e-03	3.59e-04	1.23e-03
ERBB3	Receptor tyrosine-protein kinase erbB-3	12	56473641	56497289	5.57e-02	8.57e-03	4.61e-02	4.34e-02	1.86e-02	4.60e-06	2.33e-05
PILRA	Paired immunoglobulin-like type 2 receptor alpha	7	99965153	99997719	2.08e-03	1.84e-02	3.85e-02	2.73e-02	2.57e-02	7.59e-04	3.38e-05
ADAM9	Disintegrin and metalloproteinase domain-containing protein 9	8	38854388	38962663	4.71e-04	3.42e-02	2.57e-01	3.24e-02	4.74e-02	3.59e-02	NA
VSIG10	V-set and immunoglobulin domain-containing protein 10	12	118501398	118573831	2.64e-04	5.21e-01	2.20e-02	1.54e-01	5.81e-02	5.01e-01	1.98e-01
LCN15	Lipocalin-15	9	139654086	139660707	7.69e-03	6.59e-02	1.88e-01	3.58e-02	6.23e-02	2.33e-03	4.06e-06
GRP	Gastrin-releasing peptide	18	56887400	56898006	1.45e-02	1.54e-01	6.02e-01	3.79e-02	9.34e-02	6.23e-04	1.75e-03
TNFSF13	Tumor necrosis factor ligand superfamily member 13	17	7461609	7464925	4.28e-01	3.27e-01	8.12e-02	4.95e-01	1.89e-01	1.12e-04	4.32e-04
BMP10	Bone morphogenetic protein 10	2	69092613	69098649	3.13e-01	2.35e-01	2.76e-01	1.73e-01	2.21e-01	4.12e-03	7.06e-04
ENTPD6	Ectonucleoside triphosphate diphosphohydrolase 6	20	25176329	25207365	4.07e-04	1.67e-01	2.15e-01	3.23e-01	2.21e-01	2.04e-02	4.69e-02
TEK	Angiopoietin-1 receptor	9	27109139	27230173	4.04e-04	2.18e-01	5.99e-01	1.80e-01	2.80e-01	1.12e-01	2.16e-01
GFRAL	GDNF family receptor alpha-like	6	55192267	55267291	2.46e-04	3.18e-01	5.05e-01	5.25e-01	4.43e-01	3.82e-01	6.40e-01

Table I: PWAS p-values for proteins identified by at least one method. The significance cutoff is 1×10^{-3}. The top row lists the models evaluated, while the second row indicates the training datasets used. "Combined" refers to the combined p-values from "LS-imp IGAP," "LS-imp EADB," and "AD Proxy." All models were tested on the ADSP data. p-values from the DeLIVR method trained on imputed traits that are significantly smaller than those from models trained on observed traits are highlighted.

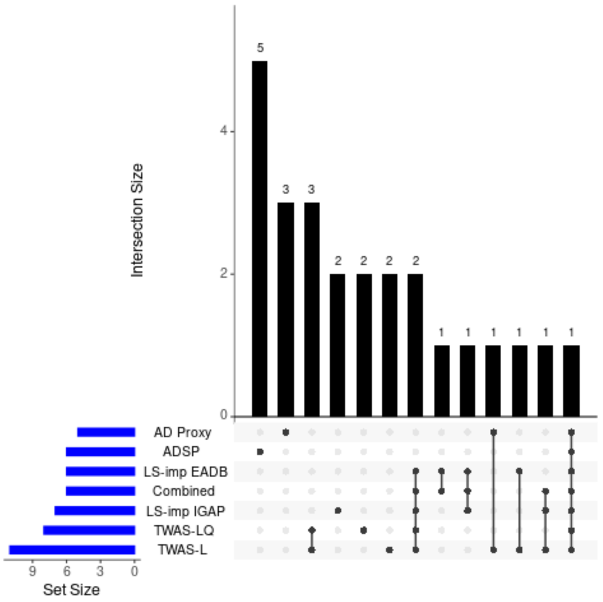

(a) UpSet plot.

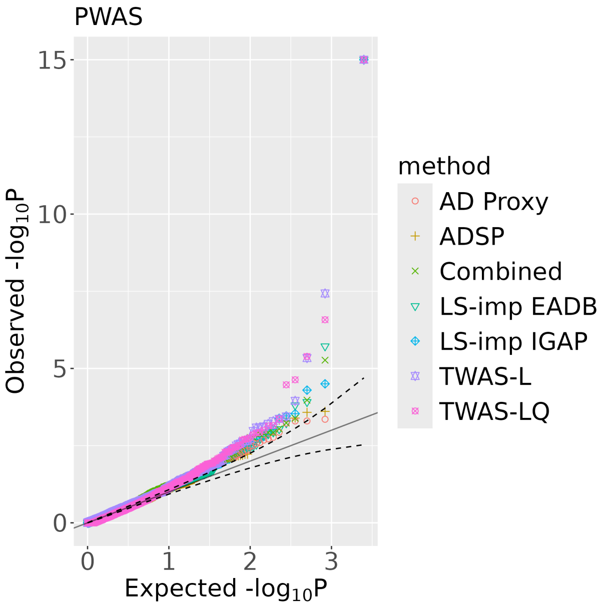

(b) Q-Q plot.

Figure R: Side-by-side UpSet plot and Q-Q plot of all models for PWAS. "ADSP" refers to the DeLIVR model trained on the ADSP data (observed AD status). "TWAS-L" and "TWAS-LQ" refer to the standard TWAS model and the parametric TWAS-LQ model trained on the ADSP data, respectively. "LS-imp EADB" and "LS-imp IGAP" refer to the DeLIVR model trained on the LS-imputed AD status using EADB and IGAP as the GWAS data, respectively. "AD Proxy" refers to the DeLIVR model trained on the proxy AD status. "Combined" refers to the results of the Cauchy combination test, which combines the p-values from "LS-imp EADB," "LS-imp IGAP," and "AD Proxy."

Model				DeLIVR					TWAS-L	TWAS-LQ
Training Data				ADSP	LS-imp IGAP	LS-imp EADB	AD Proxy	Combined	ADSP	ADSP
Gene	chr	TSS	TES							
MS4A6A	11	60184665	60184666	1.5e-02	3.7e-05	4.0e-07	2.4e-04	1.2e-06	2.2e-07	5.8e-07
CR1L	1	207645173	207645174	2.8e-03	6.4e-05	2.0e-05	2.4e-06	6.2e-06	3.8e-01	3.5e-01
FNBP1L	1	93448130	93448131	8.5e-02	1.2e-05	8.3e-06	2.6e-05	1.2e-05	4.7e-02	7.1e-03
CBS	21	43076942	43076943	5.1e-03	1.3e-03	3.5e-05	1.3e-04	8.0e-05	1.5e-03	5.3e-03
RSPH14	22	23145020	23145021	1.9e-01	3.3e-04	1.4e-04	7.8e-05	1.3e-04	1.8e-03	6.7e-03
GNAZ	22	23070360	23070361	1.6e-02	2.3e-04	1.3e-04	1.5e-04	1.6e-04	9.5e-03	NA
SESN2	1	28259526	28259527	2.7e-01	**3.6e-04**	**2.7e-04**	**2.5e-04**	**2.8e-04**	4.6e-02	1.3e-01
PLD2	17	4807095	4807096	7.3e-02	1.1e-04	1.2e-02	9.3e-04	2.8e-04	4.1e-03	1.1e-02
CYB5R3	22	42649567	42649568	5.2e-02	1.0e-01	**2.1e-04**	2.4e-02	**6.2e-04**	3.9e-02	6.2e-02
TMEM116	12	112013184	112013185	2.1e-02	4.7e-01	2.2e-04	6.6e-01	6.5e-04	1.0e-01	6.2e-04
YME1L1	10	27154822	27154823	1.4e-01	1.4e-03	1.8e-02	2.8e-04	6.9e-04	1.5e-03	5.7e-03
IPO8	12	30695985	30695986	5.3e-02	1.4e-02	1.6e-03	2.8e-04	7.0e-04	6.7e-01	NA
NECTIN2	19	44846174	44846175	7.5e-02	**4.6e-04**	5.6e-03	2.0e-03	1.0e-03	2.8e-01	5.3e-01
BLOC1S3	19	45178744	45178745	1.9e-02	4.3e-04	2.0e-02	6.4e-02	1.3e-03	1.5e-04	3.9e-04
ZNF880	19	52369916	52369917	5.4e-02	2.7e-01	6.6e-03	**4.5e-04**	1.3e-03	6.0e-01	3.3e-01
MED16	19	893217	893218	8.4e-01	**4.9e-04**	4.6e-01	8.6e-03	1.4e-03	6.2e-01	7.7e-02
RP11-536K7.3	10	5945899	5945900	1.3e-03	3.0e-03	6.1e-04	1.7e-01	1.5e-03	7.8e-01	8.2e-01
RP13-942N8.1	12	123363867	123363868	7.3e-03	6.3e-01	2.3e-03	7.7e-04	1.7e-03	5.8e-01	4.2e-01
NSUN6	10	18659284	18659285	7.7e-01	6.5e-04	8.0e-03	6.6e-02	1.8e-03	3.7e-03	3.5e-03
OSER1	20	44210790	44210791	2.7e-01	9.6e-01	1.7e-02	**6.1e-04**	1.8e-03	1.8e-01	3.9e-01
DAPK3	19	3971122	3971123	3.2e-01	1.6e-01	**6.0e-04**	7.6e-01	1.8e-03	6.9e-02	9.2e-02
SLC41A3	3	126101560	126101561	7.6e-01	4.3e-02	**7.1e-04**	1.5e-02	2.0e-03	3.9e-01	6.6e-01
CDK2AP1	12	123272333	123272334	2.6e-01	**8.2e-04**	6.9e-02	5.7e-03	2.1e-03	4.9e-02	5.4e-02
GUSBP5	4	143559471	143559472	5.7e-02	3.1e-02	1.5e-01	8.6e-04	2.5e-03	2.9e-04	1.3e-03
ZNF438	10	31031936	31031937	8.1e-01	2.0e-02	1.3e-01	**9.0e-04**	2.6e-03	4.1e-01	3.0e-01
SEL1L3	4	25863759	25863760	3.3e-02	**9.8e-04**	3.3e-02	1.6e-01	2.8e-03	2.0e-02	6.1e-02
CD27-AS1	12	6450893	6450894	8.3e-01	2.8e-03	8.3e-02	2.1e-02	7.1e-03	7.0e-04	1.0e-03
RPL23AP7	2	113627089	113627090	6.7e-04	5.0e-02	2.8e-03	4.9e-02	7.5e-03	2.6e-03	7.7e-04
ADAM15	1	155050565	155050566	5.7e-04	1.8e-02	5.0e-02	5.3e-02	3.2e-02	1.2e-02	2.9e-02
CTSH	15	78949573	78949574	7.4e-03	8.3e-01	1.6e-02	8.6e-02	4.3e-02	4.8e-04	1.5e-03
CTB-39G8.3	17	45397024	45397025	1.9e-01	2.1e-01	2.2e-02	1.3e-01	5.3e-02	9.7e-04	4.2e-03
ITGA4	2	181457206	181457207	2.0e-01	6.7e-01	2.8e-01	7.2e-01	5.6e-01	8.9e-01	9.0e-05

Table J: p-values of genes identified by at least one method. The significance cutoff is 1×10^{-3}. The top row lists the models evaluated, while the second row indicates the training datasets used. All models were tested on the ADSP data. p-values from DeLIVR trained on imputed traits are highlighted when they are significantly smaller than those from models trained on the observed trait.

Models					DeLIVR					TWAS-L	TWAS-LQ
Training Data					ADSP	LS-imp IGAP	LS-imp EADB	AD Proxy	LS-imp Combined	ADSP	ADSP
Protein name	Gene name	chr	start	end							
Apolipoprotein E	*APOE*	19	45409011	45412650	0.00e+00	0.00e+00	0.00e+00	0.00e+00	0.00e+00	2.07e-44	6.16e-45
Retinoic acid receptor responder protein 2	*RARRES2*	7	150035408	150038763	4.80e-03	**4.68e-05**	1.50e-03	**9.02e-10**	**2.71e-09**	8.33e-04	3.75e-03
Basal cell adhesion molecule	*BCAM*	19	45312328	45324673	9.62e-05	9.14e-02	5.56e-01	2.43e-09	7.30e-09	4.01e-08	6.01e-24
Apolipoprotein C-I	*APOC1*	19	45417504	45422606	2.42e-05	1.23e-07	2.38e-02	1.51e-05	3.65e-07	7.21e-07	8.79e-14
Complement receptor type 1	*CR1*	1	207669492	207813992	4.73e-03	5.21e-06	1.47e-05	1.15e-03	1.15e-05	2.00e-06	9.81e-06
Tumor necrosis factor ligand superfamily member 14	*TNFSF14*	19	6663148	6670599	1.06e-01	**2.72e-05**	8.66e-01	7.98e-03	**8.13e-05**	6.85e-01	2.81e-02
Nectin-2	*NECTIN2*	19	45349432	45392485	5.58e-02	5.60e-04	3.83e-04	5.73e-04	4.89e-04	3.80e-03	2.89e-03
Gastrotropin	*FABP6*	5	159614374	159665742	1.08e-01	3.78e-04	2.21e-03	2.42e-02	9.56e-04	3.46e-04	1.59e-03
Secretory carrier-associated membrane protein 3	*SCAMP3*	1	155225770	155232221	1.70e-02	8.60e-04	9.99e-04	1.79e-03	1.10e-03	1.06e-03	NA
Leukocyte elastase inhibitor	*SERPINB1*	6	2832566	2842240	5.12e-02	1.46e-01	**6.45e-04**	4.01e-01	1.93e-03	1.76e-01	8.52e-02
Lipocalin-15	*LCN15*	9	139654086	139660707	3.89e-03	1.50e-03	3.98e-03	2.02e-03	2.13e-03	1.59e-04	7.99e-04
Tumor necrosis factor ligand superfamily member 13	*TNFSF13*	17	7461609	7464925	2.19e-02	2.96e-01	4.84e-02	7.25e-04	2.14e-03	1.14e-04	5.81e-04
TBC1 domain family member 17	*TBC1D17*	19	50380682	50392005	8.98e-01	1.05e-02	5.62e-03	**9.88e-04**	2.33e-03	1.51e-02	5.22e-02
Carbonic anhydrase 13	*CA13*	8	86132816	86196302	3.47e-01	7.41e-03	2.77e-03	1.51e-03	2.59e-03	7.01e-04	3.27e-07
Scavenger receptor class F member 1	*SCARF1*	17	1537152	1549041	1.45e-02	1.64e-01	1.40e-01	1.95e-03	5.72e-03	4.38e-04	1.96e-03
Tubulin-specific chaperone A	*TBCA*	5	76986991	77164604	4.98e-02	1.00e-02	2.54e-02	3.48e-03	7.03e-03	8.81e-04	2.49e-04
Charged multivesicular body protein 1a	*CHMP1A*	16	89710839	89724253	9.49e-02	1.64e-02	1.04e-02	1.82e-02	1.42e-02	3.32e-03	8.44e-06
N-sulphoglucosamine sulphohydrolase	*SGSH*	17	78180515	78194722	5.41e-05	3.44e-02	9.50e-03	2.30e-02	1.69e-02	2.48e-02	1.71e-03
A disintegrin and metalloproteinase with thrombospondin motifs 13	*ADAMTS13*	9	136279478	136324508	6.51e-03	4.32e-02	7.67e-03	2.88e-01	1.92e-02	3.42e-02	2.60e-04
Chymotrypsin-like elastase family member 3A	*CELA3A*	1	22328149	22339032	6.66e-04	2.24e-01	2.76e-02	6.18e-02	5.33e-02	5.71e-01	7.15e-01
Pro-cathepsin H	*CTSH*	15	79213400	79241916	1.31e-02	8.43e-01	3.74e-02	5.02e-01	1.36e-01	4.12e-03	9.03e-04
Angiotensin-converting enzyme	*ACE*	17	61554422	61599205	1.27e-01	8.09e-01	1.09e-01	8.82e-02	1.76e-01	9.79e-04	2.84e-03
Ubiquitin/ISG15-conjugating enzyme E2 L6	*UBE2L6*	11	57319129	57335757	8.74e-04	9.86e-01	9.80e-01	9.17e-01	9.77e-01	8.56e-01	2.09e-02

Table K: p-values of significant proteins identified for Alzheimer's disease by at least one method. The significance cutoff is 1×10^{-3}. The top row lists the models evaluated, while the second row indicates the training datasets used. All models were tested on the ADSP data.
