# Supplementary material for: Enhancing nonlinear transcriptome- and proteome-wide association studies via trait imputation with applications to Alzheimer’s disease
Source: PLoS Genet. 2025 Apr 10;21(4):e1011659. doi: 10.1371/journal.pgen.1011659 (PMC12040266; doi:10.1371/journal.pgen.1011659)
Supplement: Attachment — Submitted filename: ReviewReport_PGENETICS.pdf [file pgen.1011659.s002.pdf]

Review Report: PGENETICS-D-24-00954

The paper focuses on enhancing nonlinear transcriptome- and proteome-wide association studies (TWAS/PWAS) through trait imputation, with a specific application to Alzheimer's disease (AD). The combination of trait imputation and nonlinear TWAS/PWAS presented in this paper might represent a novel application in the sense that it has not been widely explored or published extensively. However, the conceptual foundation is based on existing methods, such as LS-imputation and DeLIVR. The most innovative aspects appear to be the comparison of imputation methods in the context of nonlinear TWAS/PWAS and the use of multiple imputation strategies to enhance gene discovery.

That said, the real data analysis results do not seem to demonstrate significant improvements in identifying novel genes, as shown in Figure 3 and Tables 1-2. Additionally, the advantage of combining different imputation methods with DeLIVR is not clearly established. While the integration of these approaches represents an interesting extension of existing techniques, the paper would benefit from a more explicit demonstration of the added value and advantages that arise from this combination.

1. I find the presentation of Type I error control and power improvement in Figure 3 somewhat confusing. How can the same figure be used to demonstrate both Type I error control and power improvement? I would appreciate clarification on this point.

2. The numerical studies could be strengthened. Specifically, in Figure 3, the use of the LM method as a gold standard for Type I error control is unclear. A more rigorous evaluation of Type I errors, such as using a permutation test, would provide stronger support for the conclusions.

3. I would like to see clearer evidence or explanations of how the proposed combination of methods offers a substantial improvement over existing approaches. From the real data analysis, I do not observe a significant improvement in identifying novel genes (Figure 3, Tables 1-2). In particular, I do not see a clear advantage of combining different imputation methods with DeLIVR. It may be helpful for the authors to explore additional applications to better demonstrate the benefits of the proposed method.

4. While the study presents results for AD and HDL, it lacks a detailed simulation study that systematically evaluates the performance of the imputation methods in various scenarios. Such a study would strengthen the findings.

5. The paper assumes that imputation methods (such as LS-imputation) perform equally well across different datasets and populations. However, it does not thoroughly discuss the limitations or potential biases introduced by these assumptions, particularly for diseases like AD, which have complex genetic architectures. Additionally, the possible bias introduced by reliance on imputation is not fully explored, especially in terms of its impact on downstream analyses.

6. The comparison of DeLIVR with some parametric models is useful, but the paper does not provide an extensive evaluation against a broader set of models or methods commonly used in TWAS/PWAS. A more comprehensive comparison, especially with other machine learning approaches, would provide stronger evidence of DeLIVR's superiority.

7. The paper mentions splitting individuals into batches for imputation but does not fully address the potential sensitivity of the results to this procedure. A discussion or sensitivity analysis regarding this aspect would help assess the robustness of the method.
