# Supplementary material for: Enhancing nonlinear transcriptome- and proteome-wide association studies via trait imputation with applications to Alzheimer’s disease
Source: PLoS Genet. 2025 Apr 10;21(4):e1011659. doi: 10.1371/journal.pgen.1011659 (PMC12040266; doi:10.1371/journal.pgen.1011659)
Supplement: Attachment — Submitted filename: Response1.pdf [file pgen.1011659.s003.pdf]

Authors' one-to-one response letter

We would like to thank the reviewers for their careful reading and thoughtful comments. In the following, we address each of the comments and the concerns from the reviewers and summarize the changes we have made accordingly; the main changes are highlighted in blue in the paper (both the main text and Supplementary).

Reviewer 1:

1. *"In this paper, you use your HDL analyses to justify using case/control imputation in your AD analyses. As genetic architectures vary among traits, showing similar results with additional traits likely to be present in biobanks, e.g. height, blood pressure, etc., would be more convincing than just HDL. More concerning is your conclusions are based on a handful of significant observations in the Venn diagrams presented in Fig 3. Just because an association was found with TWAS-LQ does not mean it is a "true positive". I know defining "true positives" is a challenge, but I suggest also calculating the proportion of genes/proteins you discover that are also listed in the GWAS Catalog for a particular trait (HDL, AD, etc.) would be helpful. Additionally, you could perform your intersection analyses at a more relaxed threshold, e.g. FDR≤0.1 rather than Bonferroni, since your Venns have such small numbers."*

   The authors' response: We completely agree that it is challenging to evaluate any imputation method while being as realistic as possible; exactly because of this reason, we use UKB complete data, e.g. of HDL, as the gold standard to evaluate any imputation method. The main point is that any true signals identified using imputed traits would be more likely to be identified with more samples of observed traits, motivating our analyses of overlapping results. To address this point more thoroughly, we have added a simulation study in Supplementary

Section 4 to explicitly demonstrate Type I error control and power. The results confirm that the Type I error rate is well controlled at the nominal level. For the intersection analyses related to HDL, we have included a set of Venn diagrams in Section 3.2.2 that depict the results with FDR ≤ 0.1. These analyses reaffirm our original conclusion.

2. *"The results presented in Tables 1 and 2 seem highly correlated across models, even though only the bolded p-values reach Bonferroni adjustment for the numbers of genes or proteins tested. Indeed, many p-values miss this significance threshold, but are still low. The only unique DeLIVR result I see is CR1L in Table 1. Thus, I am not convinced the associations are unique to deep learning methods. Similar to above, if you relax thresholds, how unique are your DeLIVR results?"*

   The authors' response: First, in general, the highly correlated results are expected and support the proposed methods as complementary to each other. Second, as suggested, we have included the results for a larger cutoff of 1e-3 in Sections 3.3.2 and 3.3.3. Our analysis revealed a greater number of signals unique to the imputed traits for both TWAS and PWAS. Notably, the increase in unique signals was much more pronounced for TWAS, whereas only a few additional signals were identified for PWAS.

3. *"Along these lines, you tested just 3,880 genes (GTEx whole blood) and 1,679 proteins (UKB plasma) in your models. I'm guessing APOE was not tested in your gene expression (eQTL) models? How many known AD genes were tested in TWAS and PWAS? GTEx also has several brain tissues available that may be quite useful for AD, which I suggest including. Rather than building your own gene expression models, you could use models publicly available for PrediXcan or FUSION to test more genes in your TWAS, 13k per tissue, which may allow better assessment of potential differences between methods."*

   The authors' response: *APOE* was not tested in the gene expression model. *APOE* was not tested since it did not pass the stage 1 QC. The F-statistic of the stage 1 model was less than 10, which was the QC cutoff. Note that *APOE* also had a small R^2 and large p-value in the pre-computed stage 1 model provided by FUSION, so it would have been filtered out as well had we used the pre-computed weights from FUSION. We also included the results with different QC and stage 1

models and the brain hippocampus tissue in the Supplementary Section 5; again *APOE* still did not pass the stage 1 QC.

4. *" "LD contamination" is often a problem with TWAS results because genes linked to each other may share eQTLs (Barbeira et al 2018, Zhou et al 2020). Including chromosome and positions in your tables listing genes (and including this info for the Fig 3 results) would be helpful. In addition, colocalization or joint analyses would help zero in on the causal gene/protein just like fine mapping for SNPs."*

   The authors' response: We have included the chr and position information. At present, existing fine-mapping methods in TWAS typically assume a linear model for stage 2, which is not directly compatible with our approach using deep learning. Accounting for LD for deep learning-based models requires a multivariate approach, which is not trivial and is left for future investigation.

5. *"I thought Fig 1 was very clear, thank you. Could you add sample size and sample splitting information to Fig 2 to make it clearer? I suggest UpSet plots rather than Venn diagrams for Fig 3, especially if you end up with larger numbers."*

   The authors' response: We have added the sample size information to Figure 2. As suggested, we have used the UpSet plot format for the TWAS/PWAS analyses due to the larger number of signals and models involved.

Reviewer 2:

1. *"I find the presentation of Type I error control and power improvement in Figure 3 somewhat confusing. How can the same figure be used to demonstrate both Type I error control and power improvement? I would appreciate clarification on this point."*

   The authors' response: In general we use the results from observed traits (of larger or complete samples) as the gold standard. To evaluate Type I error rates, we compared models trained on imputed data with those trained on the complete dataset, demonstrating that the Type I error rate is comparable to models trained on the observed trait (which, as usual, is expected to have well-controlled type I errors). For power, we compared models trained on imputed data with those trained on a much smaller observed dataset to highlight the advantages of imputation. These comparisons have been clarified further in Section 3.2.2. Additionally, we have included a new simulation study in Supplementary Section 4, which confirms that the Type I error rate is well controlled at the nominal level.

2. *"The numerical studies could be strengthened. Specifically, in Figure 3, the use of the LM method as a gold standard for Type I error control is unclear. A more rigorous evaluation of Type I errors, such as using a permutation test, would provide stronger support for the conclusions."*

   The authors' response: We can't afford to run a permutation test to achieve highly significant p-values (i.e. with a large number of permutation) with computing-intensive neural network training. We have added a simulation study in Supplementary Section 4 to show the Type I error control.

3. *"I would like to see clearer evidence or explanations of how the proposed combination of methods offers a substantial improvement over existing approaches. From the real data analysis, I do not observe a significant improvement in identifying novel genes (Figure 3, Tables 1-2). In particular, I do not see a clear advantage of combining different imputation methods with DeLIVR. It may be helpful for the authors to explore additional applications to better demonstrate the benefits of the proposed method."*

The authors' response: First, our main point is not about the combination of methods, but that trait imputation can improve over using small samples of observed traits only. Second, to combine different imputation methods, we have now used the Cauchy combination test to aggregate the p-values. By the advantage of combining different imputation methods, we refer to the benefit of integrating results from multiple imputed traits rather than relying on a single imputation method. The extent of improvement from the combination test depends on the distinctiveness of the results from each model. In Supplementary Section 5, we present the results using brain hippocampus tissue, where the combination test identified the largest set of genes among all models.

4. *"While the study presents results for AD and HDL, it lacks a detailed simulation study that systematically evaluates the performance of the imputation methods in various scenarios. Such a study would strengthen the findings."*

   The authors' response: As suggested, we have added a simulation study in Supplementary Section 4 to demonstrate the Type I error control and potential power gain of using imputed traits.

5. *"The paper assumes that imputation methods (such as LS-imputation) perform equally well across different datasets and populations. However, it does not thoroughly discuss the limitations or potential biases introduced by these assumptions, particularly for diseases like AD, which have complex genetic architectures. Additionally, the possible bias introduced by reliance on imputation is not fully explored, especially in terms of its impact on downstream analyses."*

   The authors' response: Since we use the observed trait in the test data, the bias introduced in training with imputed traits is not likely to result in spurious findings as confirmed by the newly added simulation. More discussions on the limitations of the LS-imputation can be found in Ren, J., & Pan, W. (2024). Statistical inference with large-scale trait imputation. Statistics in Medicine, 43(4), 625-641.

6. *"The comparison of DeLIVR with some parametric models is useful, but the paper does not provide an extensive evaluation against a broader*

set of models or methods commonly used in TWAS/PWAS. A more comprehensive comparison, especially with other machine learning approaches, would provide stronger evidence of DeLIVR's superiority."

The authors' response: It is not the main point of the current paper to investigate the performance of DeLIVR, but the combined use of DeLIVR (or any other non-linear machine learning methods) and trait imputation. In particular, we focus on demonstrating the ability of LS-imputation to preserve potential nonlinear SNP-trait associations and it's impact on downstream analyses such as TWAS/PWAS. While DeLIVR is a flexible framework used to learn the nonlinear gene/protein-trait mapping, other methods can be potentially applied; our goal here is not to demonstrate the superiority of DeLIVR.

7. *"The paper mentions splitting individuals into batches for imputation but does not fully address the potential sensitivity of the results to this procedure. A discussion or sensitivity analysis regarding this aspect would help assess the robustness of the method."*

   The authors' response: The results for different batch sizes are included in the Supplementary (Sections 1 and 2). This problem is specific to LS-imputation, which was discussed in more details in the original LS-imputation paper by Ren et al (2023, HGG Adv).

Reviewer 3:

1. *"Please connect the comparison of marginal effect sizes, standard errors, and p-values reported in Results to the actual tables/figures in Supplementary Information. It was challenging to follow the results."*

   The authors' response: Thank you for this helpful and important suggestion. We have added the references to the Supplementary tables and figures in Section 3.1.1

2. *"How was the distribution of significant SNPs identified via LS-imputation (and the other trait imputation methods) compared to the results from the corresponding GWAS results (IGAP or EADB). The authors indicate that they "compared the Manhattan plots" of the original GWAS to the results from the trait imputation methods. There's a lot of hand-waving in this section of results and quite a bit of subjectivity."*

   The authors' response: We compared the Manhattan plots visually. We have also added Venn diagrams showing the significant SNPs identified by each GWAS analysis in Supplementary Figure 15. The Venn diagrams show that the distribution of significant SNPs identified using LS-imputed AD status closely resembled that from the corresponding GWAS results as LS-imputation had the most overlaps with the GWAS used for imputation.

3. *"Similarly for the comparison of the various imputed traits (using R2 or Nagelkerke's R2) in Results, it would be important to link this section of Results to the relevant Supplementary Information."*

   The authors' response: As suggested, we have added the references in Section 3.1.2.

4. *"I'm concerned that the TWAS did not identify the well-known Alzheimer's gene APOE, which has been consistently identified by previous TWAS studies (and implicated by previous GWAS). Now, of course, APOE was identified in the PWAS analysis (on imputed AD status). How do the authors interpret these findings?"*

   The authors' response: *APOE* was not included in the TWAS analysis due to a low stage 1 F-statistic. We have rerun the analysis with

different stage 1 SNP filters and stage 1 models. The results are in the Supplementary Section 5. But again, *APOE* did not pass the stage 1 QC (i.e., the cis genetic component of the gene expression was too weak).

5. *"Given that with the exception of APOE and APOC1, no other proteins were identified by DeLIVR across all imputation approaches, how specifically would the authors "combine" the various results? It's unclear how this combination should be done; it would be useful if the authors can make recommendations along these lines given the results presented here."*

   The authors' response: We have now used the Cauchy combination test to combine the p-values from different models. The extent of improvement from the combination test depends on the distinctiveness of the results from each model. In Supplementary Section 5, we present the results using brain hippocampus tissue, where the combination test identified the largest set of genes among all models.

6. *"The study was done using GTEx whole blood. The tissue specificity of the approach/results is unclear. (There's a strong argument to be made that whole blood is not the ideal tissue for the analysis, given the availability of brain in GTEx (and other reference resources) and given its well-recognized cell type heterogeneity.)"*

   The authors' response: We have now added the brain hippocampus tissue in the Supplementary Section 5, and reached the same conclusion.

7. *"The study started with $\geq$ 2800 proteins, but ended up with only $\leq$ 1700 proteins for the stage 2 PWAS analysis. It's unclear how many proteins were dropped at each step of the preprocessing and QC. This would be very useful information."*

   The authors' response: The only QC we applied to filter genes/proteins was based on the stage 1 F-statistic or R^2 of the expression prediction model. We have added a comment in Section 2.4.1 of the main text and Section 5 of the Supplementary to explicitly state that the genes/proteins included were those with stage 1 models passing the F-statistic or R^2 filter.

8. *"It would be important to present the Q-Q plot of the association p-values. It's important for readers to see this distribution given the control for type I error claimed here."*

   The authors' response: As suggested, the Q-Q plots are now added in Sections 3.3.2 and 3.3.3.

9. *"The authors should make all results available, including the TWAS/PWAS models used and the association results (not just the code), for ease of reproducibility."*

   The authors' response: As suggested, we have uploaded the models and results to Github.
