# Supplementary material for: Enhancing nonlinear transcriptome- and proteome-wide association studies via trait imputation with applications to Alzheimer’s disease
Source: PLoS Genet. 2025 Apr 10;21(4):e1011659. doi: 10.1371/journal.pgen.1011659 (PMC12040266; doi:10.1371/journal.pgen.1011659)
Supplement: Attachment — Submitted filename: response_letter2.pdf [file pgen.1011659.s004.pdf]

Response to Reviewers

We would like to thank the reviewers for their careful reading and thoughtful comments. In the following, we address each of the comments and the concerns from the reviewers and summarize the changes we have made accordingly; the main changes are highlighted in blue in the paper (both the main text and Supplementary).

Editor:

1. *"We would like to see the issue on type I errror raised by reviewer #2 to be addressed before making a decision."*
   The author' response: We have addressed reviewer 2's question about Type I Error rate in Section 2.1 both empirically and theoretically. We emphasize that even from **theoretical** point, there will be no inflated type I errors (under the usual assumption that **observed** data are not biased).

Journal Requirements:

1. *"Thank you for stating that "Individual-level data from the UK Biobank (UKB `https://www.ukbiobank.ac.uk/`), the Genotype-Tissue Expression (GTEx `https://gtexportal.org/home/`) Project, and the Alzheimer's Disease Sequencing Project (ADSP `https://adsp.niagads.org/`) are available by application through their respective data access processes." Please note that these links seem to direct to very general homepages rather than pages with the specific data. Please update your statement to provide further details or direct page links."*

   The author' response: Unfortunately, we do not have direct page links for the datasets as they are not publically available.

2. *"The file inventory includes files for Figures 3a, 3b,3c,3d,3e,3f ,5a,5b,6a and 6b. We would recommend either combining these into single Figure 3.tiff , Figure 5.tiff and Figure 6.tiff files with separate internal panels, or renumbering them as individual figures, as we are not able to publish multiple components of a single figure as separate files."*

   The author' response: We have combined all subfigures into a single figure.

3. *"Please ensure that the affiliations of the authors listed on the manuscript title page do exactly match with the affiliations provided in the online submission form. NOTE: Affiliations should include a department (if applicable), an institution, a city, and a country."*

   The author' response: The affiliations on the manuscript title page are correct and include a department, an institution, a city, and a country.

Reviewer 1:

1. *"The Discussion limitations have not been updated to include your brain hippocampus analysis. This analysis should be mentioned in the main text."*

   The authors' response: We have mentioned the brain hippocampus analysis in both the Results and Discussion sections.

2. *"Similarly, your response to reviewers about APOE transcript models should be included in the main text or supplement as readers will want to know this information."*

   The authors' response: We have added this information as a side note in the Results section.

3. *"Thank you for including your code in github. Please be sure to add a README to describe what scripts and results files are included."*

The authors' response: We have added a README file in the github repo.

Reviewer 2:

1. *"For my previous comments 1&2, 4 (Type I error): The authors have added additional simulations to address my previous comments. However, the type I error analysis remains unconvincing. It does not seem reasonable to use other existing methods as a gold standard to determine whether a new discovery is a false positive. For instance, the authors state that in one case (n_1 = 4000), DeLIVR Imputed uniquely identified several genes missed by all other methods, suggesting these could be potential false positives. This raises concerns about the claim that DeLIVR Imputed has higher power. As a result, this approach does not appear to provide a valid method for demonstrating control of type I error."*

   The authors' response: AT the end of Section 2.1 we have added a **theoretical** explanation about why our approach of using independent **observed** data for hypothesis testing (while only using imputed data for training) would not inflate the type I error regardless of imputation errors or uncertainty. Our simulation results listed in in Supplementary Section 4.2, Table 3 also empirically confirm this point: using imputed trait values for training and an independent observed dataset for testing can control the Type I error rate.

   Regarding the question of whether using an existing method to define true/false positives makes sense, we did not define true/false positive this way. The "existing methods" in Figure 3, e.g. "DeLIVR Complete" and "TWAS-LQ Observed", were used as benchmarks to which we compare the proposed approach, "DeLIVR Imputed". We simply call the benchmarks "gold standard" as they are based on **all observed** data and have been previously shown to control Type I error rates, and they represent an ideal scenario where all phenotypes are observed. The concerning scenario would arise if, using the proposed approach, we observed many new signals that are not identified in the ideal case, as it would be impossible to determine whether these new signals are true or false positives. However, in our study, we identified only one or few additional signals, which would not raise significant concerns.

2. *"For my previous comments 3&6: There seems to be a contradiction in the authors' responses. In response to my comment 3, the authors stated: "First, our main point is not about the combination of methods, but that trait imputation can improve over using small samples of*

observed traits only." However, in response to comment 6, they stated: "It is not the main point of the current paper to investigate the performance of DeLIVR, but the combined use of DeLIVR and trait imputation." These statements appear inconsistent, leaving me unclear about the main purpose of the paper. Please clarify the primary focus and goals in the manuscript to address this confusion."

The authors' response: In response to comment 3, the term "combination of methods" refers to combining different imputation approaches (e.g., aggregating results from LS-imputation and AD proxy methods). In contrast, in comment 6, the phrase "combined use of DeLIVR and trait imputation" refers to employing DeLIVR as the analysis model along with trait imputation by LS-imputation or proxy-AD cases. Here, "combined use" specifically denotes integrating a nonlinear analysis model with a trait imputation method that preserves nonlinear associations, which differs from combining multiple imputation models. Since the phrase "combination of models" was used with distinct meanings in these contexts, the arguments are not contradictory.

3. *"Then, in response to the author's comments to my comment 6: The presentation of the results is somewhat confusing and gives the impression that the authors are claiming DeLIVR combined with other methods is superior to TWAS-L and TWAS-LQ. However, based on the data analysis results, particularly Table 2, I still do not see a clear advantage of DeLIVR combined with other imputation methods compared to existing TWAS approaches (e.g., TWAS-L and TWAS-LQ). If this is not the intended message, please clarify this in the manuscript."*

   The authors' response: For the PWAS results (Table 2 and Figure 6), we acknowledge that TWAS-L and TWAS-LQ (referred to as the "parametric models" in the paper) identified the highest number of proteins related to AD. We did not claim that DeLIVR trained with imputed traits was superior in this case. Instead, we highlighted that DeLIVR uniquely identified certain proteins, complementing the findings from the parametric models. To clarify this point, we have revised portions of Sections 3.3.3 and 4.

   Note that in Section 4, we state that the complementary effect is supported by the observation that "aggregating results from different imputation methods yielded the largest set of genes discovered." This does not contradict our previous claim that the significance of DeLIVR lies in the unique genes/proteins it identifies rather than the total number

of genes/proteins discovered. The reason is that, in one case, we compare DeLIVR with imputed traits to the parametric models, whereas in another, we discuss the benefits of aggregating results from multiple imputation methods to capture more signals.
